# Ethical Challenges of Medicine and Health on the Internet: A Review

**DOI:** 10.2196/jmir.3.2.e23

**Published:** 2001-06-28

**Authors:** Kirsti A Dyer

**Keywords:** Internet, Ethics, Medical, Ethics, Professional, Ethics, Informatics, Physician-Patient Relation, Code of Ethics, Research Ethics, Medical Informatics Ethics

## Abstract

Knowledge and capabilities, particularly of a new technology or in a new area of study, frequently develop faster than the guidelines and principles needed for practitioners to practice ethically in the new arena; this is particularly true in medicine. The blending of medicine and healthcare with e-commerce and the Internet raises many questions involving what sort of ethical conduct should be expected by practitioners and developers of the medical Internet. Some of the early pioneers in medical and healthcare Web sites pushed the ethical boundaries with questionable, even unethical, practices. Many involved with the medical Internet are now working to reestablish patient and consumer trust by establishing guidelines to determine how the fundamentals of the medical code of ethical conduct can best be adapted for the medical/healthcare Internet. Ultimately, all those involved in the creation, maintenance, and marketing of medical and healthcare Web sites should be required to adhere to a strict code of ethical conduct, one that has been fairly determined by an impartial international organization with reasonable power to regulate the code. This code could also serve as a desirable, recognizable label-of-distinction for ethical Web sites within the medical and healthcare Internet community. One challenge for those involved with the medical and healthcare Internet will be to determine what constitutes "Medical Internet Ethics" or "Healthcare Internet Ethics," since the definition of medical ethics can vary from country to country. Therefore, the emerging field of Medical/ Healthcare Internet Ethics will require careful thought and insights from an international collection of ethicists in many contributing areas. This paper is a review of the current status of the evolving field of Medical/Healthcare Internet Ethics, including proposed definitions and identification of many diverse areas that may ultimately contribute to this multidisciplinary field. The current role that medicine and health play in the growing area of Internet communication and commerce and many of the ethical challenges raised by the Internet for the medical community are explored and some possible ways to address these ethical challenges are postulated.

## Introduction

The practice of medicine is rooted in a covenant of trust among patients, physicians, and society.

The ethic of medicine must seek to balance the physician's responsibility to each patient and the professional, collective obligation to all who need medical care.

The Council of Medical Specialty Societies, 2000 [1]

Ethics can be viewed as a prerequisite for the success of medical practice, much the same way that safety is a prerequisite for the success of airline travel. In both cases, if the prerequisites are not in place to ensure trust in the product or services provided, consumers will not utilize the product or service. In the case of the medical field, the public trusts the medical profession to regulate its own practices [2]. Knowledge and capabilities of new technology or an area of study often develop faster than the guidelines and principles needed for practitioners to practice ethically in the new arena. One area of rapid technological and economic expansion is that of the Internet, in particular how quickly the Internet is impacting and changing the practice of medicine in the 21^st^ century. We hope, for the success of medical practice, even with the rapid changes in technology and the medical field, that practitioners involved with the Medical Internet will continue to behave ethically. This paper will review the ethical challenges raised by the Internet for the medical community, explore the role that medicine and health play in the growing area of Internet communication and commerce, and postulate some possible solutions for addressing these new challenges.

## Methods

Available, published and related articles were located with an Ovid MEDLINE search for "Internet" and "Ethics, Medical," "Internet" and "Ethics, Professional." The Internet was searched for "medical and ethics," "Internet and ethics," "science and ethics," and "research and ethics" using the Google search engine (www.google.com). Additional articles and information were located by hand searching pertinent online medical journals, related organization Web sites, and relevant medical LISTSERVs: MWM-L (Medical Web Masters List), ISMHO (International Society of Mental Health Online), and AIR-L (Association of Online Researchers, AoIR).

## Results

### Background: The Integration of the Internet into Daily Life

For many of us the Internet has been integrated into our daily lives, with e-mail use becoming as commonplace as talking on the telephone. This modern method of communication has been the fastest-growing medium in the world, reaching 50 million users in only 4 years, compared to radio, which existed for 38 years before reaching 50 million listeners, and television, which took 13 years to reach the same level of use [3]. Although it is difficult to determine the exact number of people online, a reasonable estimate from Nua Internet Surveys in November 2000 was 407.1 million people worldwide, with 167.1 million in the US and Canada and 113.14 million in Europe [4].

### Background: The Integration of the Internet into Medicine

The Internet has the potential to substantially alter the way medicine is practiced, from simple e-mail communication to routine billing, distant consultations, and routine patient care. There are more than 20,000 Web sites online devoted to medicine and healthcare [5] originating from diverse sources-medical, health, personal, and commercial. Online health consumers (also known as patients) can access: Web sites related to health, on-line support groups, chatrooms and Web sites devoted to a specific disease, pharmaceutical sites, alternative-health sites, information on medical products, and online practitioners or consultants. By recent estimates, 52 million American adults, or 55% of those with Internet access, have used the Web to obtain health or medical information [6]. The number of adults using the Internet for health information, shopping for health products, and communicating with payers and their providers is anticipated to reach 88.5 million by 2005 and is projected to grow at approximately twice the rate of the overall online population [7].

The number of people surfing the Internet in search of health and medical information has not gone unnoticed by the business sector. The merger of medicine and healthcare with e-commerce has resulted in a number of online business models: selling services or healthcare products, creating high-profile health or medical portals, and providing online services to physicians and healthcare providers. Even with the recent decline in "dotcoms," there is still great projected monetary potential for those involved with medical and healthcare sites. Business-to-consumer (B2C) healthcare commerce is expected to become a $70 billion industry by 2003, while business-to-business (B2B) healthcare commerce is expected to grow into a $170 billion industry [8]. Although a recent survey conducted by Medem showed that half of all physician practices surveyed indicated that they already had or planned to build a Web site for their practice [9], there is a realistic concern that if medical and health practitioners, insurance companies, and hospitals do not provide the services demanded by their patients, then the online healthcare consumers may turn to seek online services from unlicensed, unqualified, or unprofessional providers - or disreputable sources [10].

An important challenge for the new class of ethicists - those studying the Medical Internet - will be determining the boundaries of "Medical/Healthcare Internet Ethics." Some of the boundaries needing to be defined include establishing the type of ethical conduct that could be expected from practitioners in this new medium, determining which of the existing codes of conduct could be adapted for use, and deciding which areas unique to the Internet will require the development of new ethical guidelines.

### Medical Ethics

Some physicians regard the decision to enter medicine as "a calling," similar to that seen in the clergy or in public service. This commitment to help and serve others has traditionally taken precedence over economic interests [11]. Medicine's code of ethics is considered to be far more stringent than the law. Most physicians are governed by their own internal code of ethics and more-formalized codes have been developed by professional organizations to advocate that their members behave ethically. The American Medical Association (AMA), one of the major medical organizations in the United States, established the Code of Medical Ethics for members, which has served as an ethical guideline since the mid 1840's. This code reinforces that "the primary goal of the medical profession to render service to humanity" while emphasizing that **"** reward or financial gain is a subordinate consideration and under no circumstance may physicians place their own financial interests above the welfare of their patients." [12] Additionally, in the AMA's 1995 Patient-Physician Covenant, physicians are reminded that "Physicians, as physicians, are not, and must never be, commercial entrepreneurs, gateclosers, or agents of fiscal policy that runs counter to our trust." [13] The Council of Medical Specialty Societies published their consensus statement in 1997 on the ethic of medicine, reminding physicians that "the practice of medicine is rooted in a covenant of trust among patients, physicians, and society. The ethic of medicine must seek to balance the physician's responsibility to each patient and the professional, collective obligation to all who need medical care." [1] However, codes of ethical conduct rely on self-regulation for enforcement.

Most medical and healthcare professional organizations have a code of ethical conduct established for their members, but many have not yet addressed the ethical issues of medicine, health, and the Internet. These organizations will need to adopt or establish ethical standards to guide their members in ethical conduct, in the areas of research, development, commerce, and practice on the Internet.

With the exponential expansion of the Internet, online entrepreneurs, business and medical, are trying to cash in on the projected potential of Internet commerce with healthcare services and products. Financial interests are sometimes placed above the welfare of visitors to the Web site. Depending on how one regards online site visitors - as visitors, clients, healthcare consumers, or patients - these actions can be seen to be in direct conflict with the AMA's Principles of Medical Ethics and other professional codes of conduct. Some may regard the paid-physician consultants to medical e-commerce sites as having a clear conflict of interest; others may not see it as an ethical issue. If one redefines the physician-patient relationship to be merely a provider-consumer one, then without "patients" there is no conflict of interest. However, if medical leaders do not introduce rigorous and credible conflict-of-interest rules, they risk eroding the public's trust in the profession to self-regulate [2]. Some of the early medical and healthcare Web site leaders pushed the boundaries of traditional medical ethics, so that many regarded their business practices as unethical. Ethical boundaries were stretched, even broken, in disregard for their Web site visitors, often in the name of profit. The medical profession, in the tradition of regulating its own, is striving to reestablish the public trust, by determining how traditional medical ethics can best be translated as codes of conduct for the medical and healthcare Internet.

### Medicine and Healthcare on the Internet

Medical websites, more than any other type of site on the Internet, should ensure visitors' personal privacy and prevent personal medical information, including patterns of use and interests, from being sold, purchased, or inadvertently entering the hands of marketers, employers, and insurers.

Principles Governing AMA Web Sites [14]

In stark contrast to typical e-commerce sites, intended for sales of products or services to visitors, medical and healthcare Web sites differ because the sites are frequently dedicated primarily to educating their Web site visitors. Also, with the medical or healthcare Internet the focus is on medical and healthcare interactions, transactions, and research that occur over the Internet. Another difference is the type of content obtained at a medical or healthcare site. This information is often of a very private nature and may result in life-altering and, in some situations, life-and-death decisions.

Business and computer professionals have typically not been held to the same ethical code of behavior as medical and healthcare professionals. With the merger of medicine and e-commerce, business, computer, medical and healthcare professionals are working side by side in developing online Web sites. Non-medical professionals involved in providing online medical services may be unaware of the unique standards they must adhere to when dealing with online healthcare consumers and may need to be educated about the obligation not to exploit patients or clients and to respect issues of privacy and confidentiality. Those who develop, maintain, and sell healthcare computing systems and components, including Web sites, have an ethical obligation to make patient care a primary concern [15].

Studies have shown that most adult Internet users are unaware their movements are being tracked and are also not aware of the personal information gathered about them when visiting a Web site [16]. Many Internet users believe they can visit a site anonymously and obtain information about controversial subjects such as AIDS, herpes, or suicide without anyone else ever knowing. Many may also believe that once their e-mail is deleted it is gone forever. In reality, e-mail is forever; messages are backed up and recoverable. Therefore, medical and healthcare Web sites should be following strict security measures to ensure that their site-users' personal medical information remains private and does not involuntarily enter the hands of marketers, employers, and insurers [14].

Communication technology is evolving. New technology - such as mobile phones, hand-held computers, personal device assistants (PDAs), and even wearable computer devices - is being developed. The ethical guidelines being developed for the Internet will need to have the flexibility to adapt and include future forms of telecommunication as they appear.

### Merging Fields of Study: Medicine, Ethics, Science, Computers, E-commerce...

Medical Internet Ethics includes several existing areas of study. How it is defined depends on who is viewing or experiencing the field. Describing Medical Internet Ethics is much like the parable of "The Blind Men and The Elephant" only with more people involved. In the parable, 6 blind men are asked to describe an elephant. Their descriptions of the elephant differ depending on which part of the animal was touched: side, tusk, knee, ear, trunk, or tail. Each man becomes convinced his experience and subsequent description is the only correct one. The updated fable occurs in countries around the world. People from different professions - physician, Web site designer, information technologist, marketing personnel, computer programmer, researcher, patient, consumer, ethicist, healthcare practitioner, hospital administrator, lawyer, and policy maker - are all asked to describe their ideal medical or health Web site. They are also asked to include what they would consider to be acceptable professional or business practices for the people involved with developing and creating the Web site. Their descriptions of the Web site and an acceptable code of conduct would be highly variable and strongly influenced by their professional viewpoint and motivating factors eg, financial, patient care, research, rules, and regulations.

Insights from professionals in the following diverse groups from countries around the world, should be included when defining this new interdisciplinary domain:

1. Healthcare delivery: physicians, nurses, pharmacists, healthcare professionals, and other healthcare personnel
2. Applied computing: systems developers, database managers, medical software developers, and Web administrators
3. Science and research
4. Government agencies: public-health and regulatory agencies
5. Healthcare services and e-commerce: providers of healthcare transactions conducted over the Internet
6. End users: healthcare consumers and patients
7. Healthcare organizations: insurance companies, management organizations, and societies
8. Administration and healthcare management
9. Medical ethics
10. Law

Those involved professionally with the medical or healthcare Internet have very different, and at times conflicting, motivation. Different professions and different professionals may have very different views on ethical practices. Furthermore, these views can be highly variable from country to country. It will be a challenge for Medical Internet Ethicists to reach a consensus on what constitutes "ethics" and which areas should be included in defining the field of Medical Internet Ethics. The remainder of this paper will explore some of these issues.

### Defining Medicine, Health, and Ethics on the Internet

In determining the ethics of the medical and health Internet, it is important to establish a common vocabulary. For the context of this paper, the use of the term "medical" is intended to cover the range of healthcare professions. The term "medical" or "medicine" is often used interchangeably with "health" or "healthcare." [17]

**Medical Internet Ethics** is the field existing at the intersection of medicine, ethics, and computers, but is conducted, occurs, or practiced in the new arena of the Internet. Therefore, a definition can be stated as:

Medical Internet Ethics is an emerging interdisciplinary field that considers the implications of medical knowledge utilized via the Internet, and attempts to determine the ethical guidelines under which ethical participants will practice online medicine or therapy, conduct online research, engage in medical e-commerce, and contribute to medical websites.

**Healthcare Ethics** is the term used in the description of Medical Ethics, in Encyclopedia of Ethics, to distinguish ethical principles that apply to healthcare providers - including nurses - other than physicians [18]. Thus, **Healthcare Internet Ethics** would involve the ethical principles that apply to nurses and other healthcare providers, however, in the context of this discussion, we are using "healthcare" interchangeably with "medicine" or "medical" when referring to Internet ethics.

**e-health and e-healthcare** are other terms being touted for use by the "dotcom" and e-commerce circles. Electronic health or e-health refers to all forms of electronic healthcare delivered over the Internet; these range from informational, educational, and commercial "products" to direct services offered by professionals, non-professionals, businesses, or consumers [19].

For better or for worse, the "e-health" terminology coined by the dotcom and e-commerce companies is being promoted to become the preferred term for healthcare services available through the Internet [19]. This formulated vocabulary tries to create an aura of specialized, technical, insider knowledge, one that requires high-priced consultants to help others decipher and apply, rather than using recognizable, understandable terms. If the e-commerce terminology becomes the standard for medical and health sites, there is a real concern that the primary interest is economic and commercial gain not patient care.

### Ethics and Self-regulation versus the Law and Enforced-regulation

The basis for the public's trust in a profession to self-regulate is the profession's fundamental responsibility to be concerned first and foremost with the public good.

Jerome P. Kassirer, MD [2]

When considering ethics one must also distinguish between what is considered "ethical" and what is considered "the law," since in many instances there is a fine line separating the two. Legal principles are often derived from ethical ones.

Ethics attempt to determine what is good or meritorious and which behaviors are desirable or correct in accordance with higher principles. It offers conceptual tools for evaluating and guiding moral decision making [15].

**Ethics** has been defined by Webster's as "the discipline dealing with what is good and bad, and with moral duty and obligation." [20] By comparison, laws instruct people directly on how to behave (or not to behave) under various specific circumstances. Furthermore, there are prescribed remedies or punishments for individuals who do not comply with the law.

**Law** is defined by Webster's as "a binding custom or practice of a community: a rule of conduct or action prescribed or formally recognized as binding or enforced by a controlling authority." [20]

Legal principles emphasize the practical regulation of morality, or behaviors and activities, whereas ethical principles deal with moral decisions. Many legal principles deal with the inadequacies and imperfections in human nature, compared with ethics, which looks to establish the ideal behaviors of individuals or groups. Legal practices also tend to be more affected by historical precedent, matters of definition, issues related to detectability and enforceability, and evolution of new circumstances than are ethical ones [15].

Dr. George Lundberg, editor-in-chief of Medscape, is one of the medical leaders working to define online medical ethics. He believes "the essence of professionalism is self-governance," and that the leaders of the e-health information enterprise should be the ones setting common standards for ethical behavior, not governments [21]. Self-regulation relies upon professionals upholding their personal and professional code of ethics; there are limited means of enforcing the ethical guidelines. During the early dotcom bonanza days of the Internet, there were few real standards of accountability and ethical behavior for medicine and healthcare on the Internet. Web sites and the organizations supporting them were left to regulate their own ethical behavior. In many instances esteemed medical leaders, professional organizations, and medical institutions proved to be less than exemplary ethical role models.

In a recent article, Dr. Jerome Kassirer, with Tufts University School of Medicine, examined the problem of "pseudoaccountability" where weak regulations give the appearance of setting and enforcing high standards [2]. His insights are equally applicable to the problems facing medicine and the Internet. There is concern that many of the existing codes of ethics are in actuality promoting pseudoaccountablility; they are lengthy codes of conduct crafted with technical or obfuscating language to give the impression of setting high standards, but are in reality non-enforceable. Dr. Kassirer and others do not believe that self-regulation guidelines for medical Web sites are enforceable [2]. Dr. Glenn McGee, a professor in bioethics at the University of Pennsylvania School of Medicine, described some of the early efforts as being self-inclusive and lacking objectivity, reminding us that "real peer review means thinking about and making rules with regard to conflict of interest." He believes these early efforts fell short of providing well-regulated, enforceable ethical codes [22]. There is a realistic concern that without mechanisms of enforcing ethical codes and rigorous, credible conflict-of-interest rules, the medical profession risks further eroding society's trust in their abilities [2]. One way of encouraging ethical conduct would be with a unifying ethical pledge-for all those professions involved with the Medical or Healthcare Internet, from designers, programmers, and developers to consultants, financiers, and managers-to promote internal ethical conduct.

### An Oath for Medical/Healthcare Internet Professionals?

Under the ancient tenets of the Hippocratic oath, physicians pledge to uphold the injunction *primum non noscere*(first do no harm). For physicians, nurses, and psychologists, ethical issues are often among the greatest challenges in practice. Long after their training, students in training tend to remember-and may even be influenced more by-experiences rather than factual knowledge. It is important that all professions involved in the development of the medical Internet understand, respect, and uphold the uniqueness of the physician-patient, practitioner- patient, or therapist-patient relationship. Goodman and Miller maintain that the Hippocratic injunction should apply to all those involved in Web site development as well as to the practitioners [17]. Placing patients/consumers first can be in direct conflict with the business model of generating profits for shareholders, but not placing patient's needs before profits can have serious consequences.

Recently there has been an interest in developing a Hippocratic-Oath-equivalent for scientists, computer scientists, engineers, and executives. Supporters of an Oath for scientists promote its great symbolic value to reaffirm the importance of scientist to behave ethically. A survey by the American Association for the Advancement of Science (AAAS) identified an estimated 15 to 16 oaths for scientists or engineers proposed or currently in use [23,24]. Many support the pledge initiated by the Student Pugwash Group in the United States [25]. Another oath promoted by the Institute for Social Inventions is a modified version of the Hippocratic Oath. Thus far, over a hundred eminent signatories, including 40 Nobel Prize winners and University Vice Chancellors are supporting this Hippocratic Oath equivalent for scientists, engineers, and executives [26].

As with the medical profession, the main value of an oath would be symbolic, but supporters believe it would also stimulate young scientists and professionals to reflect on the wider consequences of their field of study before embarking on a career in academia or industry [25]. Proponents believe the oath would encourage a deeper reflection by scientists and engineers on the conduct and impact of their work and create a greater sense of accountability [24]. The long-term goal is for ethics to be included in the scientific curriculum and that an ethical oath would become part of the graduation ceremony for scientists, engineers, and executives.

### Why be concerned about online ethical principles? The Not-so-Legitimate Internet Practitioners

Complicating the situation, in addition to all of the legitimate online practitioners abiding by the current ethical principles, there are also the unethical, voyeuristic people functioning outside of the traditional boundaries of medical and healthcare Internet ethics. These individuals often push the limits of Internet laws and existing Web site code of ethics, even blatantly ignoring one of the fundamentals of medical ethics whereby patients trust their physician or provider to maintain confidentiality of their personal medical information. For these unscrupulous individuals looking to make easy money on the Internet before they get caught, self-regulation may not be enough to protect healthcare consumers. The risk of losing out to the competition causes many to compromise their ethics, bending, even breaking the rules, believing that if they do not their competitors will win their market share.

Unfortunately, additional ethical guidelines would have little impact on the not-so-legitimate computer and Internet practitioners; this is where upholding internal moral-belief systems and codes of conduct may have to give way to enforceable laws. The groups outlined below - the crackers; virus and worm writers; e-paparazzi; e-stalkerazzi; online information brokers, industrial spies and unlicensed, unqualified online information providers, or online charlatans - are not bound by internal or medical Internet ethics to adhere to patient confidentiality or ensure that their Web site visitors come to no harm.

Some of The Not-So Legitimate Practitioners:

**Cracker:** malicious meddler who tries to discover sensitive information by poking around. One who breaks security on a system. Coined ca. 1985 by hackers in defense against journalistic misuse of the term "hacker," which more properly refers to the highly skilled computer programmers who enthusiastically enjoy programming and sharing their expertise [27,28].

**Virus writer:** Writer of a cracker program that searches out other programs and 'infects' them by embedding a copy of itself in them, so that they become Trojan horses (a malicious, security-breaking program that is disguised as something benign). When these programs are executed, the embedded virus is executed too, thus invisibly propagating the 'infection.' Unlike a worm, a virus cannot infect other computers without assistance [29].

**Worm writer:** writer of program that propagates itself over a network, reproducing itself as it goes. A worm can infect other computers without assistance. The term has taken on negative connotations, since it is assumed, nowadays, that only crackers write worms [30].

**e-paparazzi, e-stalkerazzi:** The Invasion of Privacy: Paparazzi bill [31] would have made it illegal to harass a person of media interest for commercial purposes including photographing, videotaping, or recording. "Cyberstalking" [32] refers to the stalker engaging in a pattern of conduct intended to follow, alarm, or harass, or making a credible threat or violating a restraining order. "Credible threat" includes threats made by means of an electronic communication device. In this context, e-paparazzi and e-stalkerazzi refers to journalists or crackers electronically gaining access to confidential information or harassing people via the Internet.

**Online information brokers:** This type of information broker is one who sells or exchanges specific information gathered on users to a Web site-often done without the users explicit knowledge. Permission is 'granted' somewhere within the fine print of the privacy policy for a Web site. These lists of specific user's demographics and preferences can be invaluable to a marketing person for targeted advertising, eg, online or mail.

**Industrial Spies:** So far, this has been primarily a part of high tech industrial espionage, in which the industrial "secret agents" obtain access to other companies' computer databases looking for company secrets to utilize or share.

**Unlicensed, Unqualified Online Information Providers, or Online Charlatans** : These non-professionals and Web entrepreneurs have flooded the Internet, offering "mentoring" or "counseling" services, "miracle cures" or other life-enhancing products. Many are working outside the ethical and legal boundaries on the Internet hawking their "life lesson" expertise, selling services or products that may constitute health fraud.

These unlicensed, unqualified online information providers are not professionals so there are no overseeing professional regulatory organizations to which they can be reported for professional misconduct. One potential solution in the United States is to report Internet fraud, to the Internet Fraud Complaint Center (IFCC), a government agency that addresses issues of fraud committed over the Internet including both criminal and civil violations [33]. The online charlatans can also be reported directly online to the Federal Trade Commission, [34] which along with the Food and Drug Administration is waging war against Internet health fraud under "Operation Cure.All." [35]

### Cybercrimes and the Medical Internet

The Internet has provided a new arena for the criminal element as well. In the US the Criminal Division's Computer Crime and Intellectual Property Section (CCIPS) was established as a separate section of the Department of Justice. Their staffs focus exclusively on the issues raised by computer and intellectual property crime, encryption, electronic privacy laws, search and seizure of computers, e-commerce, cracker investigations, and intellectual property crimes [36].

With the growth of the Internet and the increase in cybercrime, it is easy to see why protection of privacy is an issue of great concern particularly among Internet users seeking health information [37]. In August 2000, it was made public that the Kaiser Permanente Health Care System had the confidentiality of 858 members breached; this security issue was actually an internal problem due to human error, not from external hackers [38]. In January 2001, the University of Washington Medical Center affirmed that a hacker had infiltrated its computer system in December 2000. The 25-year-old hacker gained access to administrative databases containing confidential records of at least 5,000 patients. Representatives from the University of Washington have since admitted that their databases were not secure [39]. The potential for further breaches is enormous. In a March 12, 2001 survey released by the Computer Security Institute and the FBI's (Federal Bureau of Investigation's) San Francisco Computer Crime Squad, they reported that 85% percent of respondents (primarily large corporations and government agencies) had detected computer security breaches within the last 12 months and 40% had detected system penetration from the outside [40]. It may only be a matter of time before the medical and healthcare professions are more affected.

One can imagine potential disastrous scenarios, both personal and professional, that could occur if medical information or a person's personal seemingly "anonymous" online health surfing habits, e-mail messages, or confidential medical records were made public. Insurance companies could hire online medical information brokers to obtain medical information on policyholders and use this information to deny coverage or claims. Potential employers could use information brokers to obtain health information and health-Web-site (eg, cancer, AIDS, herpes, suicide, alcohol, and depression Web sites) surfing habits on current or potential employees and use this information to fire or not employ a person. Online marketers are already using private information to design targeted e-mail advertising that fills our e-mail boxes. The potential risks for celebrities and others in the public eye may be even higher. With the premium attached to getting "unauthorized" photographs of public figures to satisfy an ever-more voyeuristic society, it is easy to imagine scenarios in which the e-paparazzi and e-stalkerazzi could be looking for, or even be paid to search for, confidential medical information on celebrities, politicians, athletes, and other prominent public figures that could be published in print and/or online media.

### Other Examples of Questionable Online Conduct

#### Cyberplagiarism

Other issues of unethical online conduct were brought to the attention of the medical and research professions by the Journal of Medical Internet Research - that of online plagiarism or cyberplagiarism. It is easy to copy and paste bits and pieces from different articles or graphics on different Web sites to "write" a paper. Cyberplagiarism occurs when a scientist or researcher "intentionally or inadvertently, is taking information, phrases, or thoughts from the World Wide Web (WWW) and using it in a scholarly article without attributing the origin." Eysenbach cautions that the only sure, reliable way of avoiding plagiarism charges is to "cite the source properly, even if it is 'only' an electronic document." Because JMIR editors were impacted personally by incidents of cyberplagiarism, the Journal of Medical Internet Research became the first scholarly journal worldwide to institute an anti-cyberplagiarism policy whereby every submitted manuscript is now electronically scanned for plagiarism [41].

#### Copyright infringement

Contrary to popular belief, just because something is available on the Internet, it does not mean anyone can use it. This applies to all of the various media forms- including text, images, and music. One has to carefully look at copyright issues, especially at what constitutes a copyrighted work, what constitutes copyright violation, and what constitutes "fair use."

The principle of "fair use" is often cited when materials found on the Internet are incorporated into lectures, scientific reviews, or education-based Web sites. Fair use of a copyrighted work can be cited when using works for the purposes of criticism, comment, news reporting, teaching, scholarship, and research. If a work falls into the category of fair use, it is exempted from normal copyright laws, and using the material is not considered an infringement of copyright. Copyright owners are required by law to consent to fair use of their works by others. Most lectures that incorporate slides or video clips into their presentations would probably fall under the category of "fair use" if the sources were being used for the purposes of "teaching, criticism, or scholarship"; however credit should be given to the source. The copyright laws are not clear as to whether using a cartoon for humor or using a music or video clip as an entertainment break in the lecture qualities as fair use [42,43].

The Internet allows users to access information from the Web across national boundaries; this creates problems when the existing laws only apply to a particular country. There is no "international copyright" that automatically protects works on the World Wide Web. Most countries do offer protection to foreign works under certain conditions specified by international copyright treaties and conventions. An international consensus will need to be reached about how these conditions apply to the Internet so that online works will be protected against unauthorized use [42].

One novel way of dealing with copyright infringement and cyberplagiarism, particularly in the scientific and research realm, is to make articles and resources freely available on the Internet and avoid copyright concerns altogether. This approach will be adopted by Massachusetts Institute of Technology with the creation of their OpenCourseWare Web site which will be available in 2002 [44] and is already in use at the University of Pittsburgh's Department of Public Health with their Supercourse [45].

### Major Areas of Medical Internet Ethics

In this emerging field of Medical/Healthcare Internet Ethics, there are at least 6 identified areas that will require codes of ethical conduct to be established. The following lists major areas and the subsequent discussion will undoubtedly stimulate thought for additional areas to include [46].

- Doctor-patient, provider-patient, therapist-client relationships
- Online medicine, online therapy
- Online research
- Quality of information on medical and healthcare Web sites
- Ethical conduct of medical and healthcare Web sites
- Privacy and security

### Defining the Essence of the Doctor-Patient, Provider-Patient, Therapist-Client Relationships

Growing numbers of patients are going online and becoming savvy healthcare consumers desiring more online contact with their physicians. Concern about the liabilities of practicing online has been a driving force to try and clearly define the online physician-patient relationship.

Online physicians and therapists are innovators, exploring the types of interactions and services that can be provided over the Internet. Thus far, a consensus has yet to be reached regarding what ethical responsibility exists, if any, between the physician or therapist and Web site visitor. One of the early pioneers in health Web sites, Dr. C. Everett Koop, felt no professional ethical obligation towards visitors to his Web site, because they were not "his patients." [47] Key questions still need to be answered to define the online relationship: Does a patient have to be seen, examined or spoken to, to have a relationship with their physician? Does a physician consultant to a Web site have an ethical obligation to visitors? Is it dependent on the type of services or contact offered to users at a Web site or the consultant's position with the Web site? What are the boundaries for an online therapist? Can traditional therapy be translated to the Internet, or is it primarily a new form-e-therapy? At what point is there a patient-provider relationship? Case law has not determined at what point, if any, the physician-patient or therapist-client relationship begins, when the only contact is between them is online.

The eRisk Working Group in Healthcare (comprised of the leading medical malpractice insurers, Medem, and medical societies) is working on determining the issues and liabilities associated with online physician-patient interactions. The AMA Council on Judicial and Ethical Affairs (CEJA) is also working to determine what constitutes the essential elements of physician-patient relationships and how this may be translated to the Internet [22]. These issues are still ill defined since there have been few legal cases challenging online physician-patient communication and cybermedicine [9]. Once these basic components of the relationship are defined, then determining guidelines for online medicine and therapy can proceed.

### Establishing Guidelines and Regulations for Practicing Online Medicine and Online Therapy

In addition to defining what constitutes an online relationship, the types of services and products that can and should be provided over the Internet will need to be clearly determined along with standards of professional online conduct.

The mental health community has been leading the efforts to define and determine the therapeutic benefits of online relationships. Seeman and Seeman have examined e-psychiatry, how the patient-psychiatrist relationship is practiced in the electronic age [48]. In January 2000 the International Society for Mental Health On-line (ISMHO), and the Psychiatric Society for Informatics (PSI) endorsed "Principles for the Online Provision of Mental Health Services" defining the online client-therapist relationship and what constitutes providing online mental health services [49]. The National Board of Certified Counselors (NBCC) has established standards for their counselors that define what constitutes an ethical practice of Web counseling [50]. Organizations in the medical community are exploring the nature of the physician-patient relationship [9,22,51].

Professionals-physicians, psychologists, psychiatrists, and social workers-are licensed by their respective professional agencies and therefore required to follow a certain professional code of conduct established by their professional boards. But what sort of training or license should be required, if any, to practice online? The logistics of who is traveling through cyberspace to meet whom is the first issue that needs to be determined. With telemedicine, many states already require licensure in their state before an out-of-state physician can electronically provide services to patients [52]. With the Internet, it needs to be decided if it is the patient or the provider traveling to meet the other through cyberspace. If the patient is traveling to meet the provider, the consensus reached in e-psychiatry, [48] then the provider is already licensed in the state where he/she practices and would not need a license. However, if the provider is the one traveling to meet the patient, following the telemedicine statutes, then the provider would need to be licensed in the state the patient was residing, severely limiting the practice of cybermedicine, e-psychiatry, or e-therapy.

How the practice of online medicine and therapy will be conducted is yet another issue. Several approaches have been proposed for credentialing practitioners of online medicine and therapy. These include: self-regulation through abiding by ethical guidelines, advanced training for "e-providers," or requiring a special license. However, self-regulation will only work if there exist some enforceable penalties for violators, otherwise the unscrupulous will push the limits hoping to make their fortune before they are caught. Additionally, special training programs for healthcare providers who want to become "e-providers" and practice online medicine or online therapy could be instituted. These programs could educate potential online providers about online ethical obligations, essentials of the provider-healthcare consumer relationship, e-commerce, privacy and security issues, and Internet legalities [46]. A more comprehensive approach would be the establishment of an independent, international body to assess "cyberdocs," issue a special license to practice in cyberspace, and then monitor their practice [53].

### Establishing Ethical Standards for Internet Research

The new forms of communication available on the Internet - chatrooms, message boards, and LISTSERVs - have created a researcher's paradise of new online arenas to utilize and study. A wide range of research tools - including online experimental studies, surveys, interviews, field observations and participant observations - are being put to use to determining how online individuals and groups are utilizing and behaving in this new media [54]. Internet users can participate in unique pilot programs such as the National Cancer Institute's LiveHelp, that provide real-time site guidance with an information specialist who will answer questions and provide direction to helpful information on the site [55].

Real-time chatrooms are a new unique method of communicating unlike any previous form of communication. This new form of communication blurs the distinction between what is "private" and what is "public" in online communities. Researchers have tried using the analogy of a "public square" to described a chatroom, but chatrooms are unusual; they are part telephone party line, part instant journaling, part random anonymous phone calls, and part permanent "recording" of Internet chat in the form of e-mail messages. Many researchers have already conducted studies to monitor discussion groups collecting research data surreptitiously as "lurkers," believing that this behavior is acceptable as long as they do not identify subjects in research projects. But is it ethical? Are researchers obligated to disclose their presence if they are collecting data by "observing" in chatrooms, knowing their presence may alter participant's behavior? Is it ethical for researchers to gather information for research projects under the guise of "naturalistic observations" from chatrooms without participants being aware they are part of a study? Does merely participating in public forums imply informed consent? Offline research projects are required to adhere to clearly delineated ethical guidelines when dealing with research participants [56]. It is essential that Internet researchers demonstrate clear ethical judgment and follow clear ethical guidelines to protect chatroom participants' privacy.

Not everything improves when moved onto the Internet. Many of the traditional research techniques do not adapt well in this new media. The anonymity of the Internet makes it difficult to get a truly randomized, unbiased study population for an online survey; anonymity and the ease of use of pseudonyms blurs key demographic details normally important to research studies such as age, gender, ethnicity, and country of origin. Online researchers face the challenge of determining which parts of the traditional research methods can be adopted or adapted for use on the Internet and which will need to be discarded as non-adaptable [46].

Several organizations have worked or are working to establish ethical standards for research and expanding them to include research on the Internet. In November 1997, the American Psychological Association (APA) issued a statement for their members to provide guidelines for dealing with services provided by telephone, teleconferencing, and the Internet [57]. The Association of Internet Researchers (AoIR) is currently working to establish guidelines for conducting online research. The American Medical Informatics Association (AMIA) is also exploring various aspects of research and informatics through their working groups - Clinical Trials and the Ethical, Legal and Social Issues (ELSI) [58,59]. The determinations of these organizations will help determine the ethical guidelines for conducting online research, which can then be combined into a cohesive set of guidelines.

### Determining Guidelines for Quality Medical and Healthcare Information on the Internet

For patients to feel confident about the medical and health information they obtain at a Web site, a standard set of ethical guidelines needs to be adopted and enforced. Many different organizations are using varied approaches to try to determine ethical guidelines for the Internet [60].

The predominant Internet - industry and public - policy approach to addressing these concerns is to encourage voluntary codes of conduct and industry self-regulation or self-governance [61]. The Health on the Net Foundation (HON) developed one of the first codes of conduct set of principles, the Net Code of Conduct [62]. The British Healthcare Internet Association has published Quality Standards for Medical Publishing on the Web [63] and an Internet Bill of Rights for Access to Health Information on the Net [64]. Editors of the Journal of the American Medical Association proposed guidelines for "assessing, controlling, and assuring the quality of medical information on the Internet." [65] Dr. George Lundberg expanded the definition of Medical Internet Ethics to include medical ethics, journalism ethics, business ethics, and the ethics of medical editing [21]. In addition to medicine, if one expands the definition and includes business and journalism ethics, other professional organizations and their codes include: the Society of Professional Journalists' Code of Ethics [66] the American Health Information Management Association's (AHIMA) recommendations to ensure privacy and quality of personal health information on the Internet, [67] and the International Committee of Medical Journal Editors' (ICMJE) policy statement about publishing on the Web [68]. The major limitation to this approach is that self-regulation does not deter the unscrupulous, who most need to have their ethical standards raised.

A second approach relies upon healthcare consumers to evaluate Web sites for quality using a checklist or rating tool. Different rating tools are available to consumers - if they know where to find them. The Health Summit Working Group of the Health Information Technology Institute of Mitretek Systems developed a Web-based, interactive Information Quality (IQ) tool for use in assessing the quality of health information on the Internet [69]. DISCERN is a brief online questionnaire, developed by the University of Oxford's Division of Public Health and Primary Health Care, at the Institute of Health Sciences. This questionnaire provides Internet users with a valid and reliable way of assessing the quality of consumer health information [70]. The Quick Web site tool, developed by the Health Education Authority and the Centre for Health Information Quality, is designed to be used as a teaching aid for children in an educational setting [71]. However, the checklist approach requires the consumer to be motivated enough to seek out, understand, and then use the rating tools.

A third approach utilizes third-party reviewers - physicians, academicians, nurses, librarians, and other experts - to evaluate health information and write reviews or create useful lists of sites, so that users, patient or physician, can determine the quality of information at these sites [60]. Review sites can be libraries such as NOAH (New York Online access to Health) [72] or MedWeb; [73] university-based or university-sponsored such as Netwellness [74], InteliHealth, [75] or Mayoclinic.com [76]; or non-profit-based such as Medical Matrix [77]. The reviewer approach is very labor intensive and dependent on the frequency that reviewed materials are updated, and requires frequent updates.

Fee-based rating or "accreditation" systems for medical and health Web sites are also being established. In May 2001, URAC ("American Accreditation HealthCare Commission," see www.urac.org, to explain the discrepancy in their name [78]) and Hi-Ethics announced that they would be collaborating on the URAC Health Website Accreditation program as a way for health Web sites to demonstrate their compliance with ethical standards. URAC is in the final stages of developing and testing its accreditation standards for health Web sites with implementation of the fee-based program scheduled to begin in August 2001. This accreditation program will involve an onsite review and analysis of Web site documentation and operations [79]. One concern with implementing fee-based accreditation systems is that this system favors the larger, well-funded organizations. URAC's proposed fee structure may exceed the yearly operating budget for many of the medical and health information Web sites. There is concern that the presence of seals of approval and certifications may provide a false sense of security and mislead consumers unless there is a system of enforcement and rigorous verification [61].

A next-generation approach is being developed by MedCERTAIN (MedPICS Certification and Rating of Trustworthy Health Information on the Net). This project is developing a self-rating and third-party rating system enabling individuals, organizations, associations, societies, and others to filter health information and identify and select high-quality information. The MedCERTAIN consortium will also establish an international trustmark for health information by creating different levels of certification for those who publish health information on the Internet. Web sites wanting the MedCERTAIN certification will have to commit themselves to the eHealth Code of Ethics [80].

### Requiring Ethical Conduct for Medical and Healthcare Web sites

Online ethics of commercial medical Web sites and the ability of the online healthcare industry to effectively self-regulate grabbed the limelight in the fall of 1999 after several prominent medical and health Web sites showed questionable ethical behavior. Among the complaints were that the distinction between objective information and advertising or promotional content was hazy and that business ties were not properly disclosed [47]. Other questionable practices included non-disclosure of business partnerships, [81] cookies tracking unsuspecting visitors, [82] and blatant conflicts of interest, with officers profiting from insider stock trading [83]. Since then, efforts to create codes of ethics for Web-based medical and healthcare activities have intensified.

One of the first codes of conduct for health and medical Web sites was developed by the HON in 1996 [62]. The following year the APA's Ethics Committee created guidelines for their members for dealing with services provided by telephone, teleconferencing, and the Internet [57]. In September 1999, Medscape published their advertising and sponsorship policy, "The Ethics of the Medical Internet." [21] During 2000, the AMA published guidelines for their medical and health information sites on the Internet to follow, [14] the Internet Healthcare Coalition's (IHC) eHealth Ethics Initiative published an International Code of Ethics, [84] the MedCERTAIN consortium published a statement of purpose and the Consensus Recommendations on Trustmarks, [85] and Health Internet Ethics' (Hi-Ethics) published Ethical Principles for the Health Internet [86]. Many of the most-trafficked health Web sites (America Online, Discoveryhealth.com, drkoop.com, Healtheon/WebMD, InteliHealth, Mediconsult/Physician's Online, and Medscape) agreed to be compliant with the Hi-ethics principles [87].

Baur and Doering of the US Department of Health and Human Services tried to make some sense of the different frameworks. They reviewed the four main private-sector proposals-HON, AMA, IHC, and Hi-ethics-and compiled a side-by-side comparison of the key elements for improving the quality of health Web sites. They found that the various codes may have different audiences and different purposes, with different motivations for developing the framework, yet all are being promoted to the general public as ways of improving quality [61].

The current framework situation is thus a bit chaotic, with many redundant, overlapping, and competing organizations. Each organization has spent a great deal of time, resources, and money to become the definitive ethical standard setting association [61]. With over 60 different instruments for rating Web sites found by Jadad and Gagliardi, [88] and a variety of proposals and ethical codes having been drafted by various profit, non-profit, and e-health organizations, it may prove to be difficult to provide Internet users with one quality rating system. Reaching a consensus does not necessarily mean merging all frameworks into one, but it does mean forging an agreement on what Web site developers and users need to do, how the information will be described, and how the guidelines will be enforced [61]. There is still no single, unifying consensus for determining quality of Web sites and establishing medical Internet ethical principles, but there is movement in the right direction, ie, movement to consolidate efforts. One can be optimistic that the agreement reached by several organizations at recent meetings and conferences is an indication of integrating efforts towards finally adopting *one* common, cohesive Medical/Healthcare Internet Code of Ethics, guaranteed by a trusted third party, that all online medical and healthcare Web sites can finally agree upon, implement, and enforce.

### Ensuring Internet Users Privacy and Security

In this age of expanding access to information, a critical ethical responsibility is recognizing the right to privacy. A considerable challenge arises from trying to balance the desire to make information freely available to users of the Internet, while at the same time protecting people's privacy and confidentiality [14]. Additionally, personal information is being transmitted to different medical and health organizations via the Internet and should be protected from intentionally or unintentionally reaching unsecured or unauthorized users. Understandably, protection of privacy is an issue of great concern among Internet users seeking health information [37].

There is an obvious need for secure Web sites, to ensure visitors' personal privacy and prevent personal medical information, including patterns of use and interests, from being sold, purchased, or inadvertently entering the hands of marketers, employers, and insurers [14]. Former US President Bill Clinton noted that "Nothing is more private than someone's medical or psychiatric records. And, therefore, if we are to make freedom fully meaningful in the Information Age, when most of our stuff is on some computer somewhere, we have to protect the privacy of individual health records." [89]

The United States has several governmental agencies responsible for certain regulatory efforts on the Internet. The Department of Health and Human Services will work to protect the confidentiality of medical records and ensure online privacy under the Health Insurance Portability and Accountability Act (HIPAA) of 1996, now scheduled to be implemented over the next few years. HIPAA will govern the privacy of medical records and protection of digital information about patients, and will require providers, claims clearing houses, and health plans to implement administrative and technical steps to protect the confidentiality of electronic health records [39]. The final law may still be amended from the original. One could envision an expanded role for the Criminal Division's Computer Crime and Intellectual Property Section to aid in the prevention of cybercrimes within the medical field. In regards to medical data, the anti-paparazzi law [31] could be extended to allow victims to sue for damages that occur from a reporter (or online information broker) obtaining personal medical information. The cyberstalking laws [32] could be expanded to include obtaining medical information via the Internet. If adapted, both laws would go a long way toward preventing private medical information from getting into the wrong hands [61]. However, even if more U.S. regulations are enacted, they are only enforceable in the US. What will happen when problems occur in the international Web community? Ultimately, online activities and behaviors may require an impartial, unifying, international regulatory body to enact and enforce international ethical regulations, and codify medical/healthcare Internet conduct.

## Discussion

### A Field in Evolution

The rapid technologic development of the Internet has opened communication and commerce to the wired world, to people and professionals in different countries with different customs, beliefs, and definitions of ethics. The Internet is also changing how medicine will be practiced in the 21^st^ century. The Internet raises many new ethical challenges for the medical community, especially when trying to consolidate different views from different countries on medical ethical practices.

Medical/Healthcare Internet Ethics is an emerging multidisciplinary field at the intersection of medicine, ethics, and the Internet. In this paper 10 diverse areas were identified that will be melded to produce the new field. These include: healthcare delivery; applied computing; science and research; government agencies; healthcare services; end users: consumers and patients; healthcare organizations: insurance companies, management organizations, and societies; healthcare management organizations; administration and healthcare management; medical Ethics; and law. Other areas will most likely be added later. In this paper Medical/Healthcare Internet Ethics was defined as "an emerging interdisciplinary field that considers the implications of medical knowledge utilized via the Internet, and attempts to determine the ethical guidelines under which ethical participants will practice online medicine or therapy, conduct online research, engage in medical e-commerce and contribute to medical Web sites."

Medical/Healthcare Internet Ethicists will be looking to current views of medical ethics and codes of professional conduct from participating countries to establish the ideal behaviors and ethical conduct for all the professions involved with the medical Internet. The areas identified for further examination and study in this paper include:

- How visitors' privacy, security, and confidentiality should be ensured when visiting a Web site or conducting transactions over the Internet.
- How Web-site visitors can determine the quality of information at a Web site.
- How the doctor-patient, patient-provider, and therapist-client relationships should be translated into practicing online medicine and online therapy.
- How Web site designers, developers, managers, and sponsors should develop and maintain ethical medical and healthcare Web sites.
- How online medical and healthcare businesses should be ethically conducted.
- How online research should be ethically conducted.
- How all the professions involved in the medical or healthcare Internet should ethically comport themselves.

The field of medicine has traditionally relied upon self-regulation of its members, especially in the area of medical ethics. However, unethical conduct by some of the early medical and health Web pioneers left both the public and medical ethicists wondering about the effectiveness of self-regulation. Although the essence of medical professionalism is self-governance, there is no way of enforcing standards if practitioners choose not to follow professional ethical guidelines, or if non-professionals have no professional guidelines to follow. There is concern that many of the existing codes of ethics developed for the Internet are in actuality promoting pseudoaccountablility, with lengthy codes of conduct crafted in technical language that convey the impression of setting high standards, but in reality are non-enforceable.

Several challenges await the practitioners, scientists, researchers, developers, programmers, patients, administrators, governments, e-commerce marketers, managers, and ethicists involved in this emerging field. An early challenge will be blending the varied definitions of "medical ethics" from many different countries into a cohesive consensus. Another will be determining which components of the existing medical, scientific, computer, management, and economic areas, among others, should contribute to defining the field of Medical Internet Ethics. How to credential practitioners interested in online medicine or online therapy - whether requiring additional training, certification, or even a special license - is yet another challenge. One key challenge will be in fully restoring the public's trust in medical and healthcare Web sites. This challenge may be solved with a cohesive code of ethics and Web site guidelines to effectively regulate the medical/health Internet industry.

Unfortunately, the Internet has many unscrupulous people and professions functioning outside of the traditional realm of medical ethics, often pushing the limits of the existing Web site codes of ethics and the Internet laws. Additional ethical guidelines would have little impact on these not-so-legitimate computer and Internet practitioners. In order to protect healthcare consumers' privacy and confidentiality, the self-regulation of ethical codes of conduct may have to evolve into enforceable laws. Without some international agreements, national regulatory efforts are only enforceable within the country that has passed the law. The worldwide availability of online locations makes it easy for unethical medical and healthcare entrepreneurs to establish their Internet company in the most permissive jurisdiction they can find, moving if necessary to another online locale to continue their Internet misconduct. Ultimately all those professionals involved in the creation, maintenance, and marketing of medical and healthcare Web sites should be required to adhere to a strict code of ethical conduct, one that has been fairly determined by an impartial international organization with reasonable power to regulate the code.

Contributing to this issue is the projected changing demographic profile of the Internet. Much of the discussion on Medical Internet Ethics has been initiated by organizations and companies from the United States or the EU (European Union). With tremendous growth of Internet use in Asia, China, and Japan, it is predicted that by 2002 the majority of Internet users will be non-English speaking [90] This changing face of the Internet further underscores the need for an international approach when developing a medical Internet regulatory organization.

Many of the international organizations - the United Nations (UN), particularly UNESCO (United Nations Educational, Scientific, and Cultural Organization), the World Health Organization (WHO), the World Trade Organization and the International Telecommunications Union - have been in the forefront of determining ethical and regulatory questions relating to: quality of information on the Internet, telemedicine, and e-commerce [91]. The past successes of the WHO and UNESCO suggest these two organizations may be well suited to unify the many disparate initiatives in Medical/Healthcare Internet Ethics. For example, the adoption of the UN-sponsored ebXML Internet communications standard confirmed that the United Nations can be an effective catalyst for standard-setting in the crucial area of Internet development [92]. The United Nations, with its specialized agencies and nonaligned consensus groups is uniquely qualified to lead discussions on Medical Internet Ethics, and perhaps establish something like a UN Commission for the Medical Internet. Such a body would be ideally suited for establishing, and most importantly, regulating, a single code of Medical Internet Ethics that would include advertising, health fraud detection, and ensuring consumer privacy [91]. The prestige afforded the United Nations would provide the authority to regulate; the prospect of being "blacklisted" by a UN Commission as an unethical medical or healthcare Web site would be a powerful deterrent to any would-be charlatan, organization, or company, when trust and public opinion is critical to a Web site's success. Additionally, UN authorities would be in the best position to gain the attention of the necessary national authorities if it became necessary to press for action against Internet-based medical or healthcare activities that endangered the health of individuals.

Many organizations from the fields of medicine, informatics, counseling, journalism, business, research, and management are already carefully deliberating to establish guidelines for their members in ethical conduct - including ethical research and ethical online practice - and translating or adapting many of the traditional codes of ethics to the Internet. Non-medical professionals involved in providing online medical services (designers, writers, backers, programmers, promoters, and executives of medical and healthcare Web sites) must be educated as ancillary healthcare professionals, so as not to exploit online patients or clients. These organizations involved in medicine and healthcare on the Internet will need to establish strong internal protection, privacy, and security measures, to ensure the safety of stored personal data and the confidentiality of transmitted information over the Internet.

In this paper, several of the key challenges have been presented and explored to stimulate more thought by the medical Internet community. The greatest challenge for all concerned with Medical Internet Ethics will be to catch up to the explosion in Internet technology and determine the most effective use of new technology in medicine and healthcare, while not compromising the fundamentals of medical ethics.

## Abbreviations

AAAS: American Association for the Advancement of Science
AMA: American Medical Association
AMIA: American Medical Informatics Association
AoIR: Association of Internet Researchers
APA: American Psychological Association
CCIPS: Criminal Division's Computer Crime and Intellectual Property Section
CEJA: Council on Judicial and Ethical Affairs of the AMA
ELSI: Ethical, Legal and Social Issues working group of the AMIA
EU: European Union
FBI: Federal Bureau of Investigation
HIPAA: Health Insurance Portability and Accountability Act
HON: Health on the Net Foundation
IFCC: Internet Fraud Complaint Center
IHC: Internet Healthcare Coalition's
ISMHO: International Society for Mental Health On-line
MedCERTAIN: MedPICS Certification and Rating of Trustworthy Health Information on the Net
NBCC: National Board of Certified Counselors
NOAH: New York Online access to Health
PDA: personal device assistant
PSI: Psychiatric Society for Informatics
UN: United Nations
UNESCO: United Nations Educational, Scientific, and Cultural Organization
URAC: American Accreditation HealthCare Commission
WHO: World Health Organization

## Multimedia Appendix

Downloadable slides of the presentation "Medical Internet Ethics: A Field in Evolution", presented at Medinfo'01, Sept 2001, London [46] ]
