# Supplementary material for: Ethical Challenges of Medicine and Health on the Internet: A Review
Source: J Med Internet Res. 2001 Jun 28;3(2):e23. doi: 10.2196/jmir.3.2.e23 (PMC1761893; doi:10.2196/jmir.3.2.e23)
Supplement: Supplementary file 1 [file jmir_v3i2e23_app1.ppt]

## Slide 1
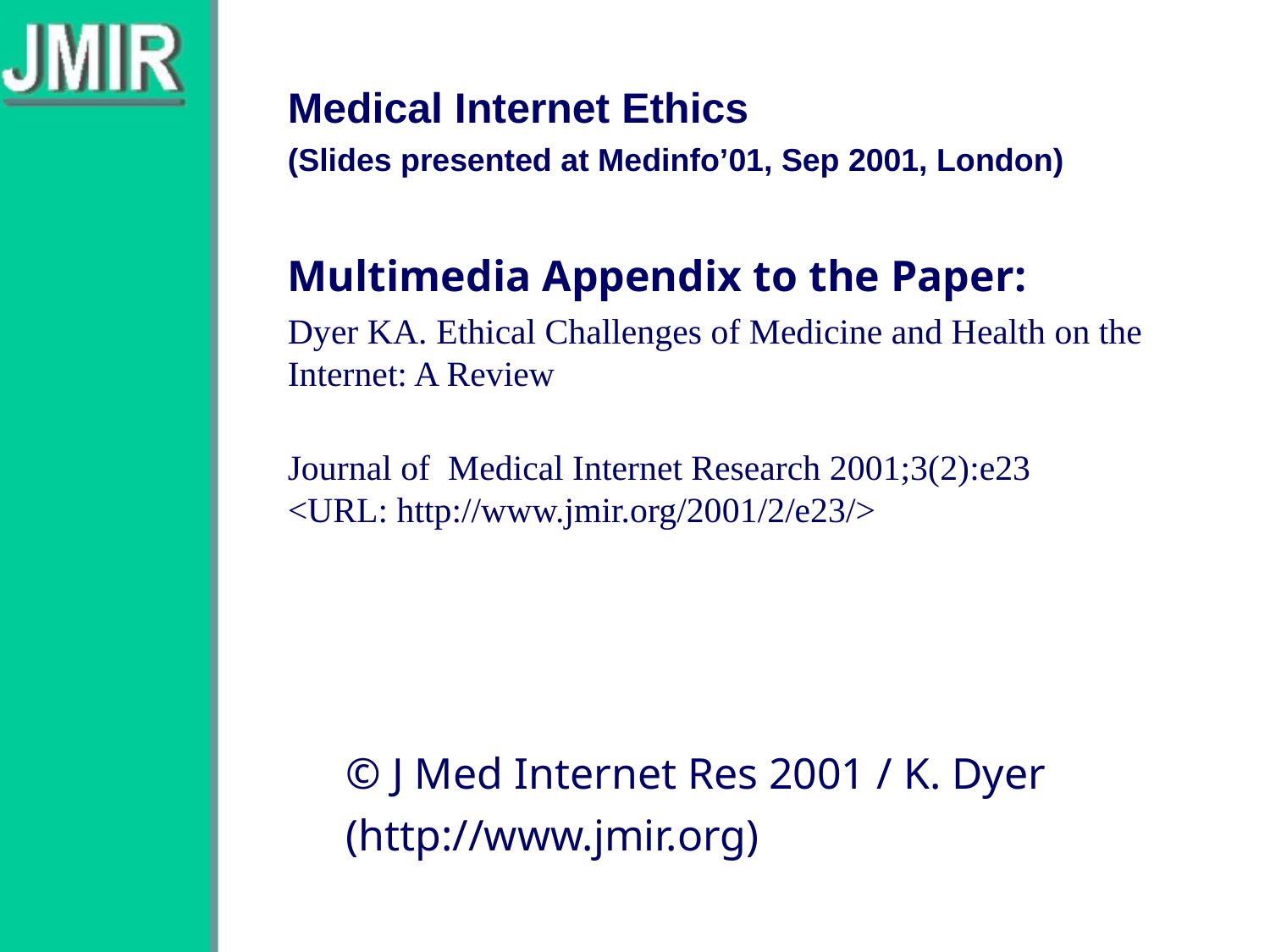

# Medical Internet Ethics
(Slides presented at Medinfo’01, Sep 2001, London)
Multimedia Appendix to the Paper:
Dyer KA. Ethical Challenges of Medicine and Health on the Internet: A Review
Journal of  Medical Internet Research 2001;3(2):e23
<URL: http://www.jmir.org/2001/2/e23/>
© J Med Internet Res 2001 / K. Dyer
(http://www.jmir.org)

## Slide 2
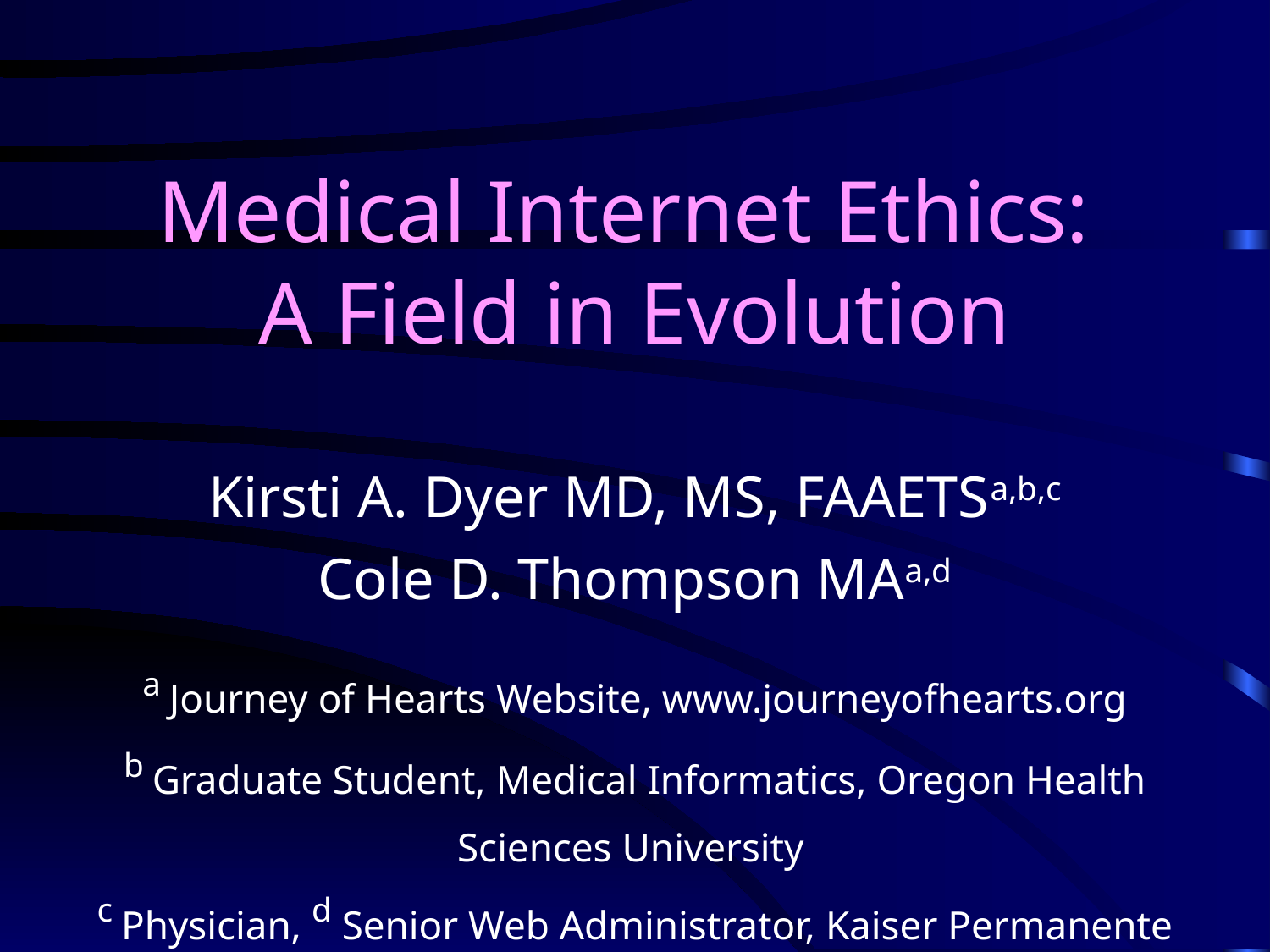

# Medical Internet Ethics: A Field in Evolution
Kirsti A. Dyer MD, MS, FAAETSa,b,c
Cole D. Thompson MAa,d
a Journey of Hearts Website, www.journeyofhearts.org
b Graduate Student, Medical Informatics, Oregon Health Sciences University
c Physician, d Senior Web Administrator, Kaiser Permanente

## Slide 3
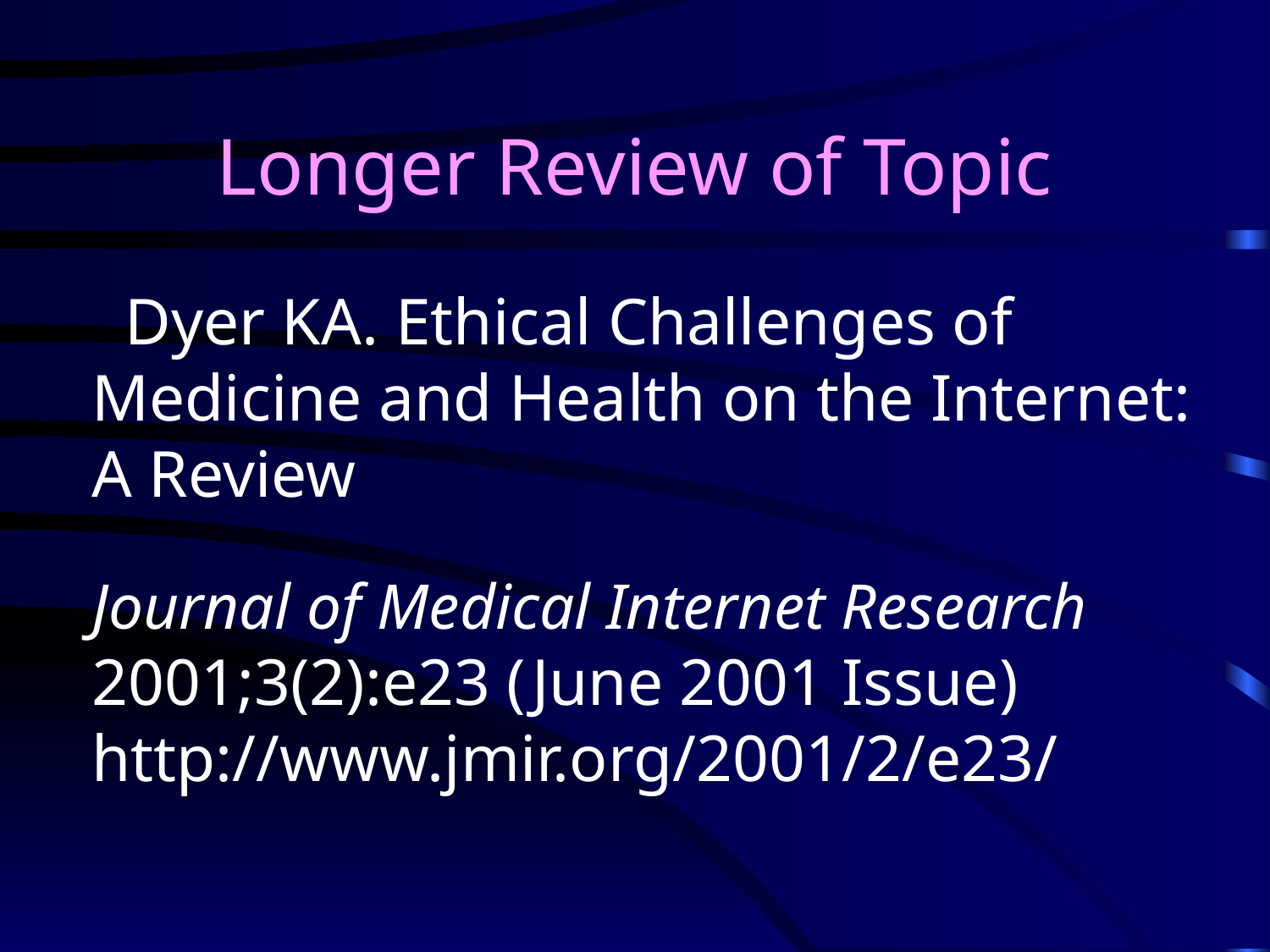

# Longer Review of Topic
 Dyer KA. Ethical Challenges of Medicine and Health on the Internet: A Review
Journal of Medical Internet Research 2001;3(2):e23 (June 2001 Issue)
http://www.jmir.org/2001/2/e23/

## Slide 4
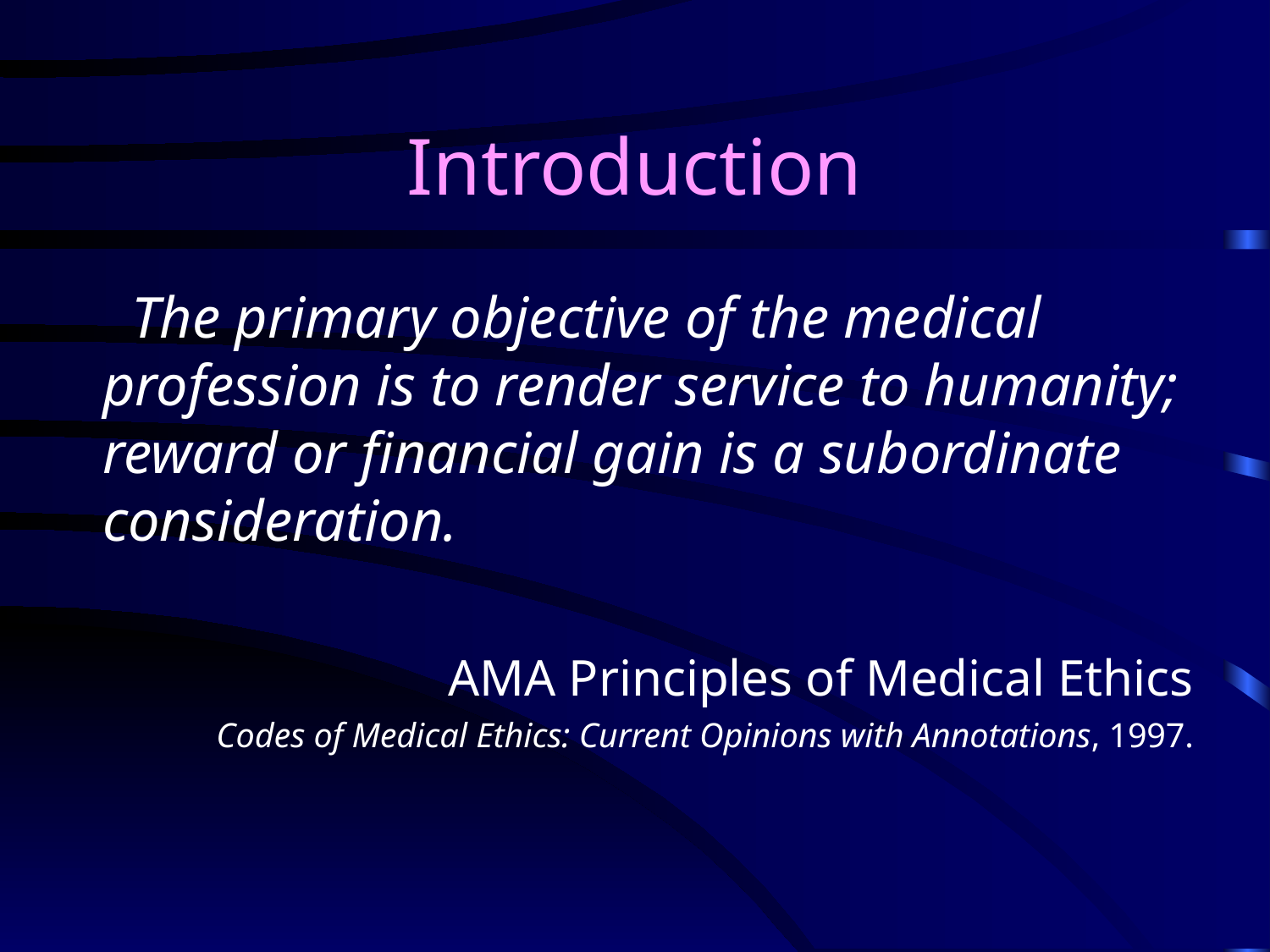

# Introduction
 The primary objective of the medical profession is to render service to humanity; reward or financial gain is a subordinate consideration.
AMA Principles of Medical Ethics
Codes of Medical Ethics: Current Opinions with Annotations, 1997.

## Slide 5
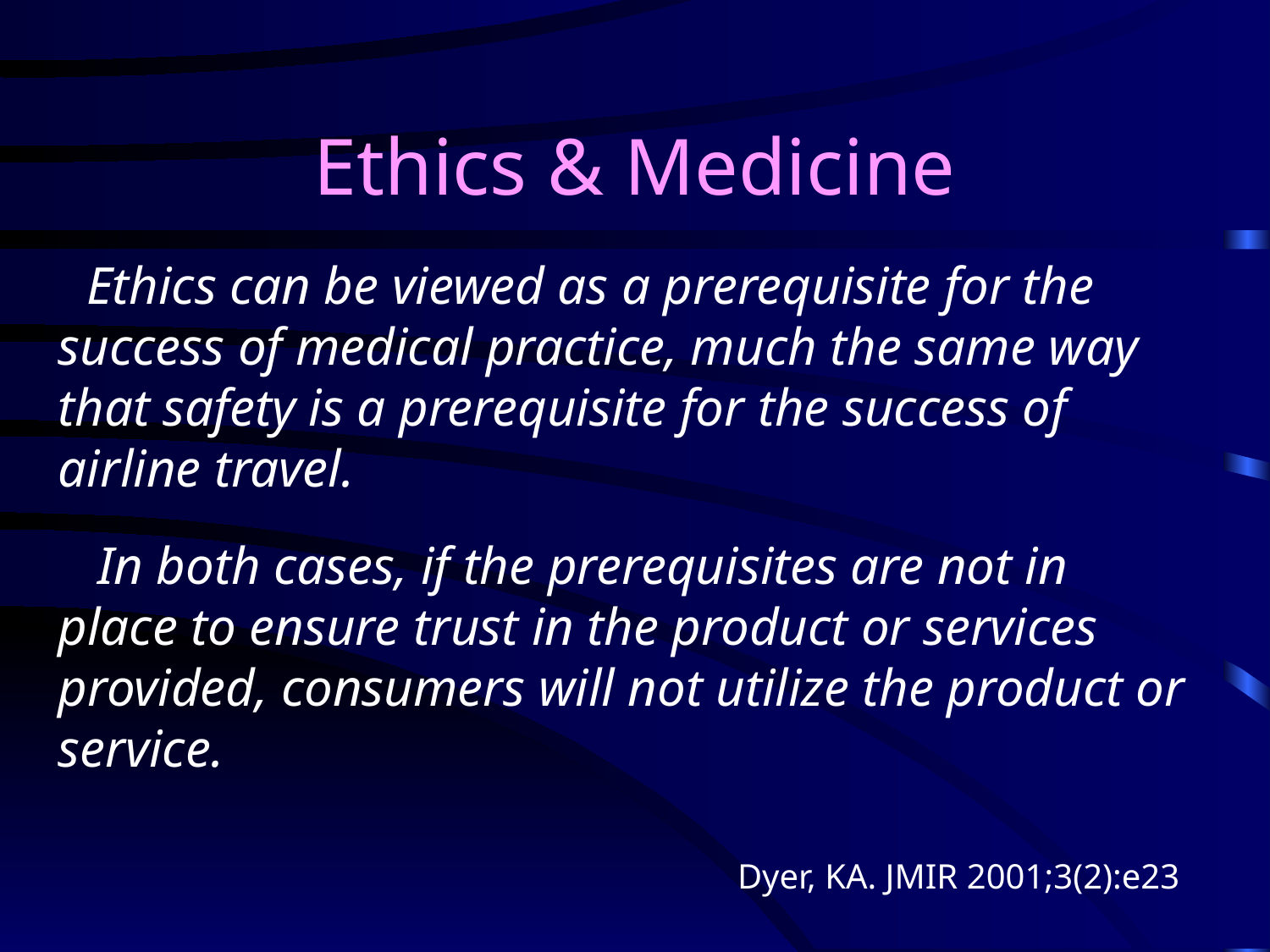

Ethics & Medicine
# Ethics can be viewed as a prerequisite for the success of medical practice, much the same way that safety is a prerequisite for the success of airline travel.
 In both cases, if the prerequisites are not in place to ensure trust in the product or services provided, consumers will not utilize the product or service.
Dyer, KA. JMIR 2001;3(2):e23

## Slide 6
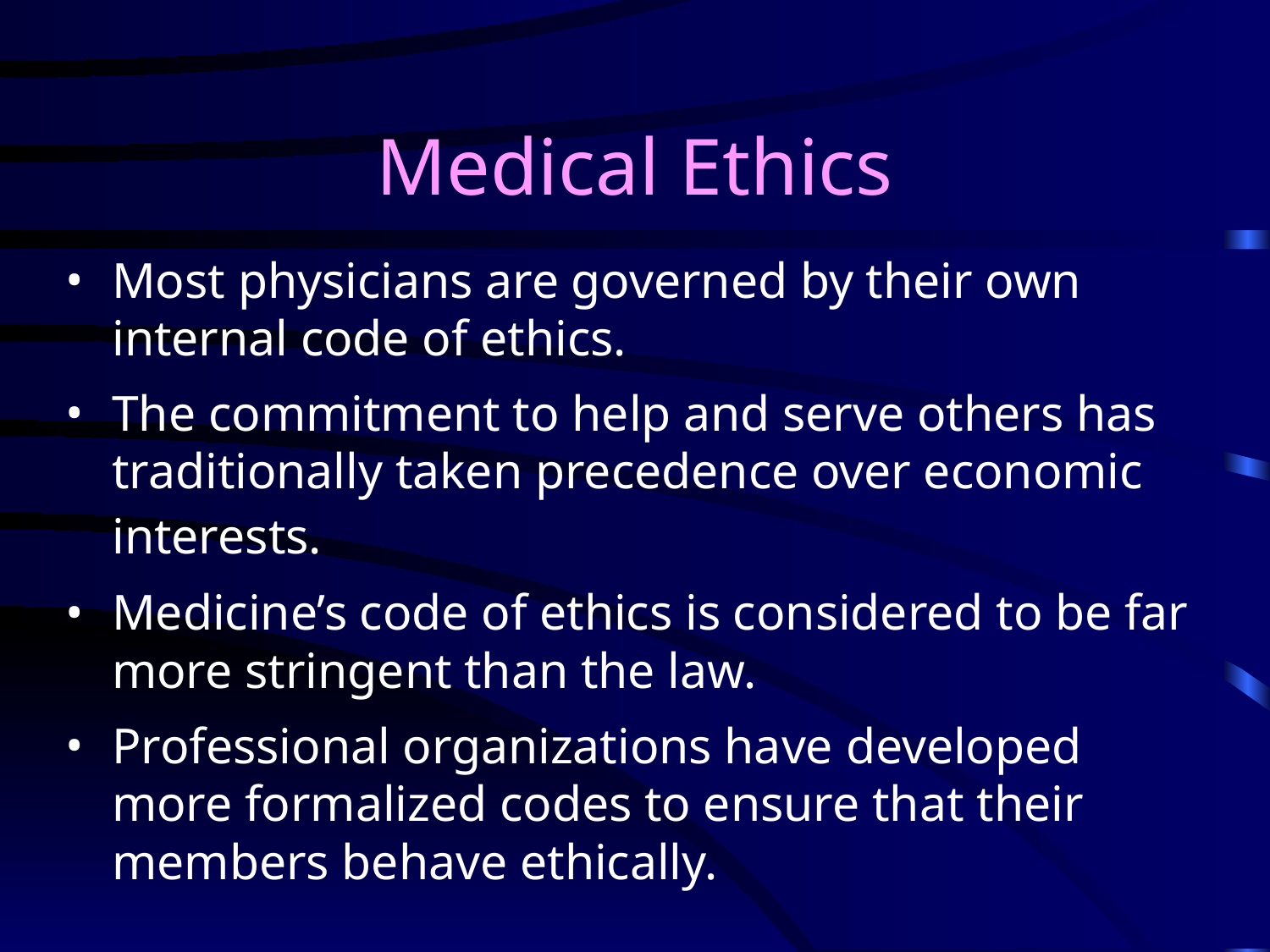

# Medical Ethics
Most physicians are governed by their own internal code of ethics.
The commitment to help and serve others has traditionally taken precedence over economic interests.
Medicine’s code of ethics is considered to be far more stringent than the law.
Professional organizations have developed more formalized codes to ensure that their members behave ethically.

## Slide 7
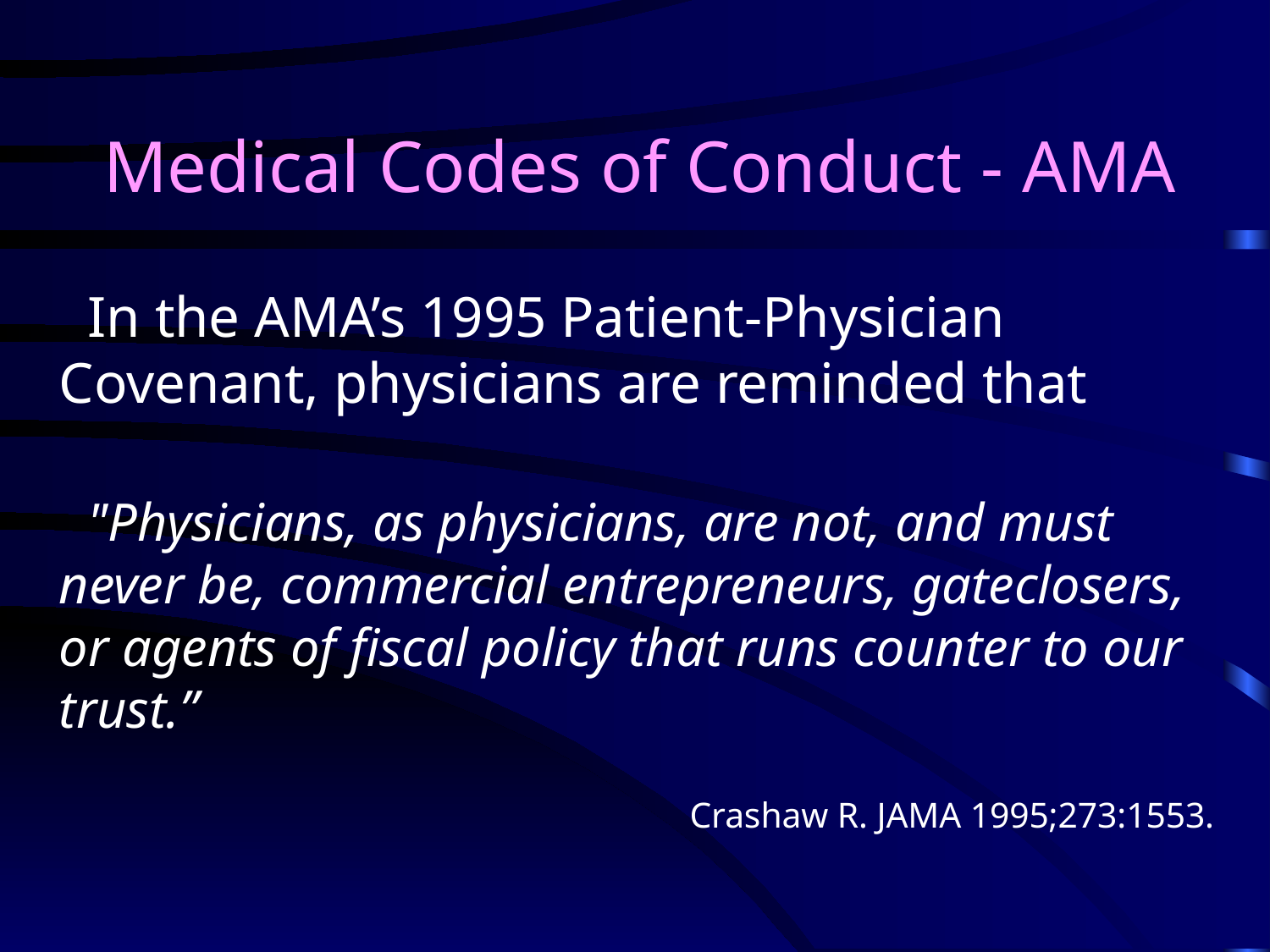

# Medical Codes of Conduct - AMA
 In the AMA’s 1995 Patient-Physician Covenant, physicians are reminded that
 "Physicians, as physicians, are not, and must never be, commercial entrepreneurs, gateclosers, or agents of fiscal policy that runs counter to our trust.”
Crashaw R. JAMA 1995;273:1553.

## Slide 8
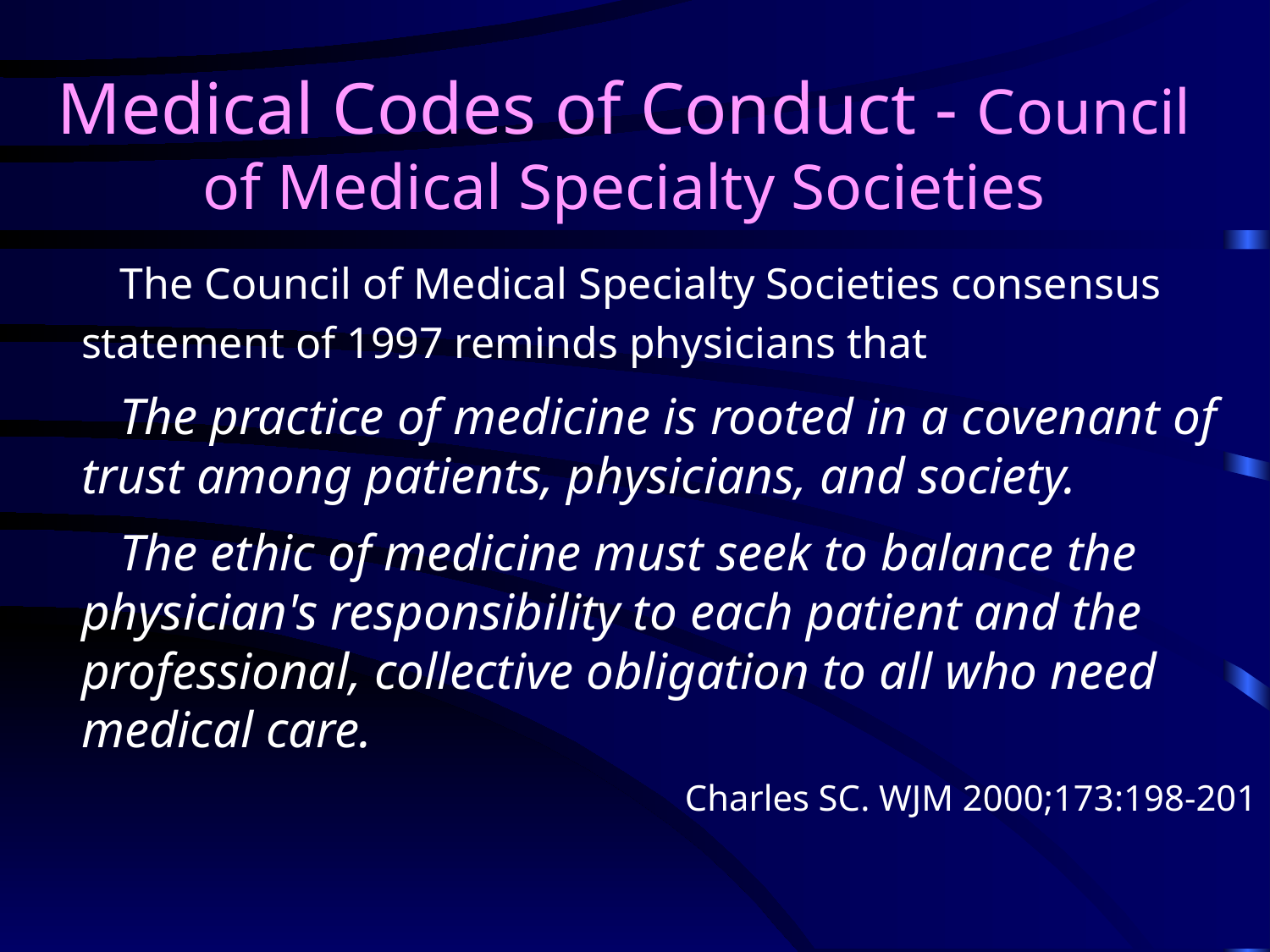

# Medical Codes of Conduct - Council of Medical Specialty Societies
 The Council of Medical Specialty Societies consensus statement of 1997 reminds physicians that
 The practice of medicine is rooted in a covenant of trust among patients, physicians, and society.
 The ethic of medicine must seek to balance the physician's responsibility to each patient and the professional, collective obligation to all who need medical care.
Charles SC. WJM 2000;173:198-201

## Slide 9
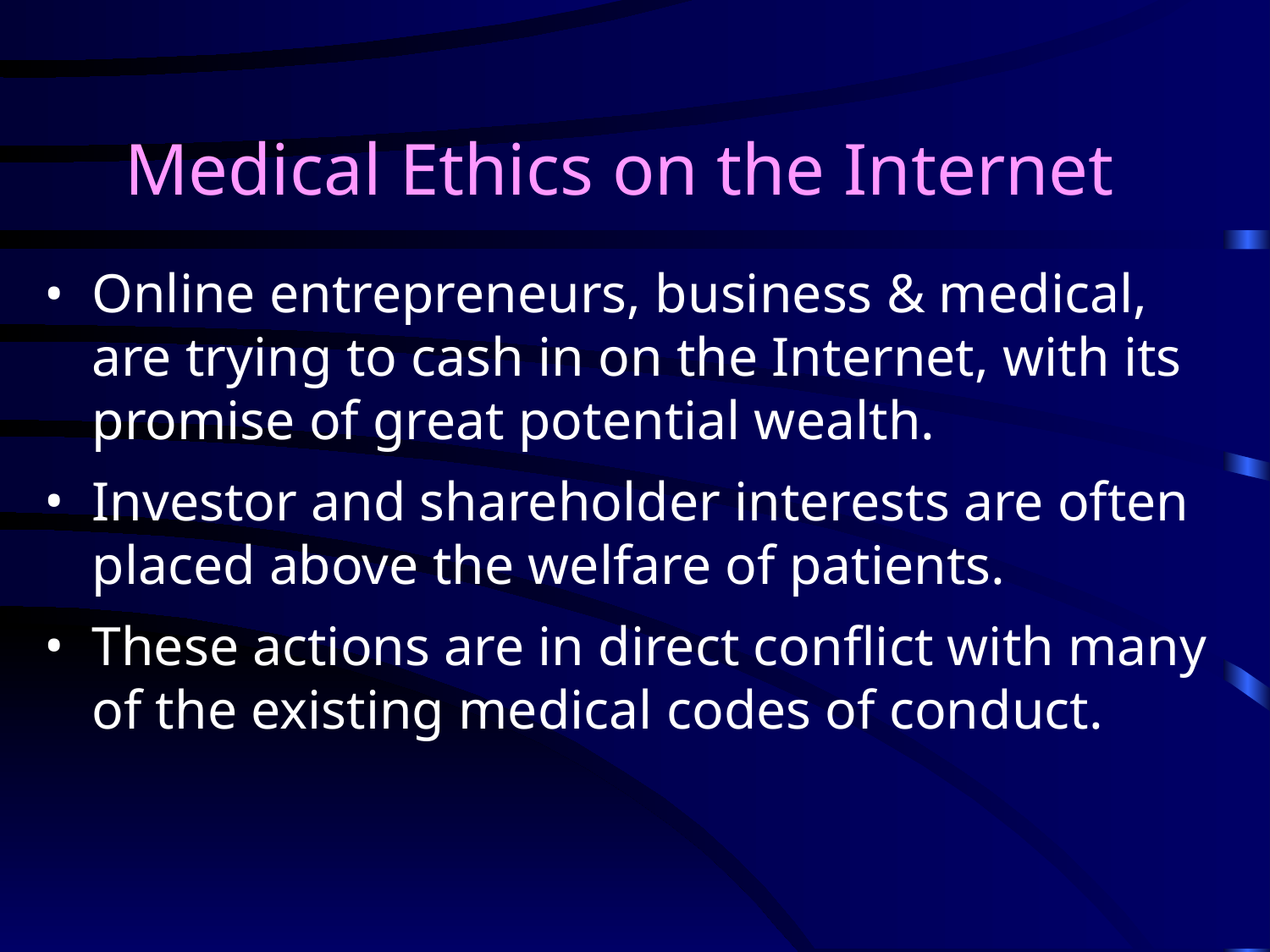

# Medical Ethics on the Internet
Online entrepreneurs, business & medical, are trying to cash in on the Internet, with its promise of great potential wealth.
Investor and shareholder interests are often placed above the welfare of patients.
These actions are in direct conflict with many of the existing medical codes of conduct.

## Slide 10
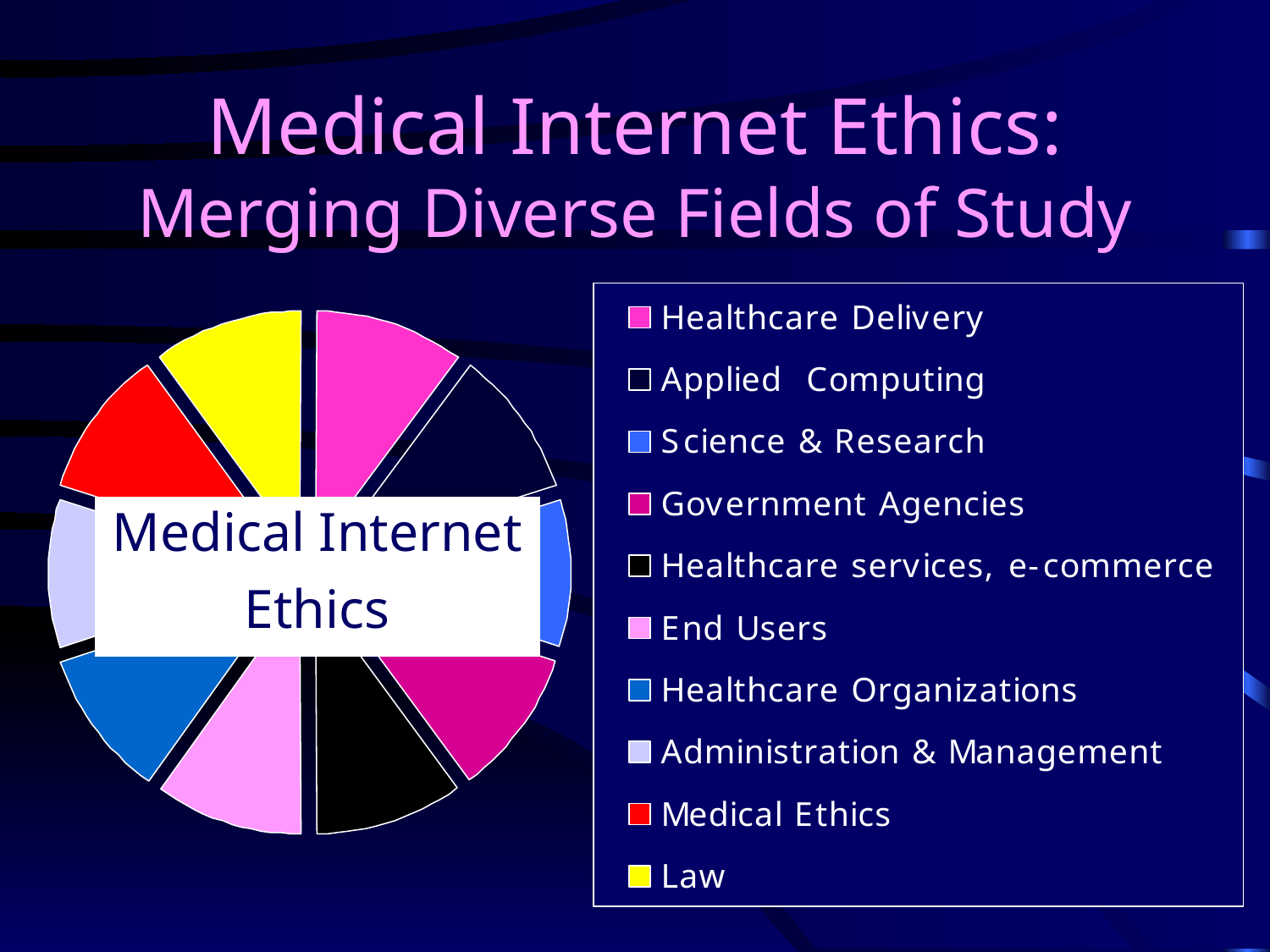

# Medical Internet Ethics: Merging Diverse Fields of Study
Medical Internet
Ethics

## Slide 11
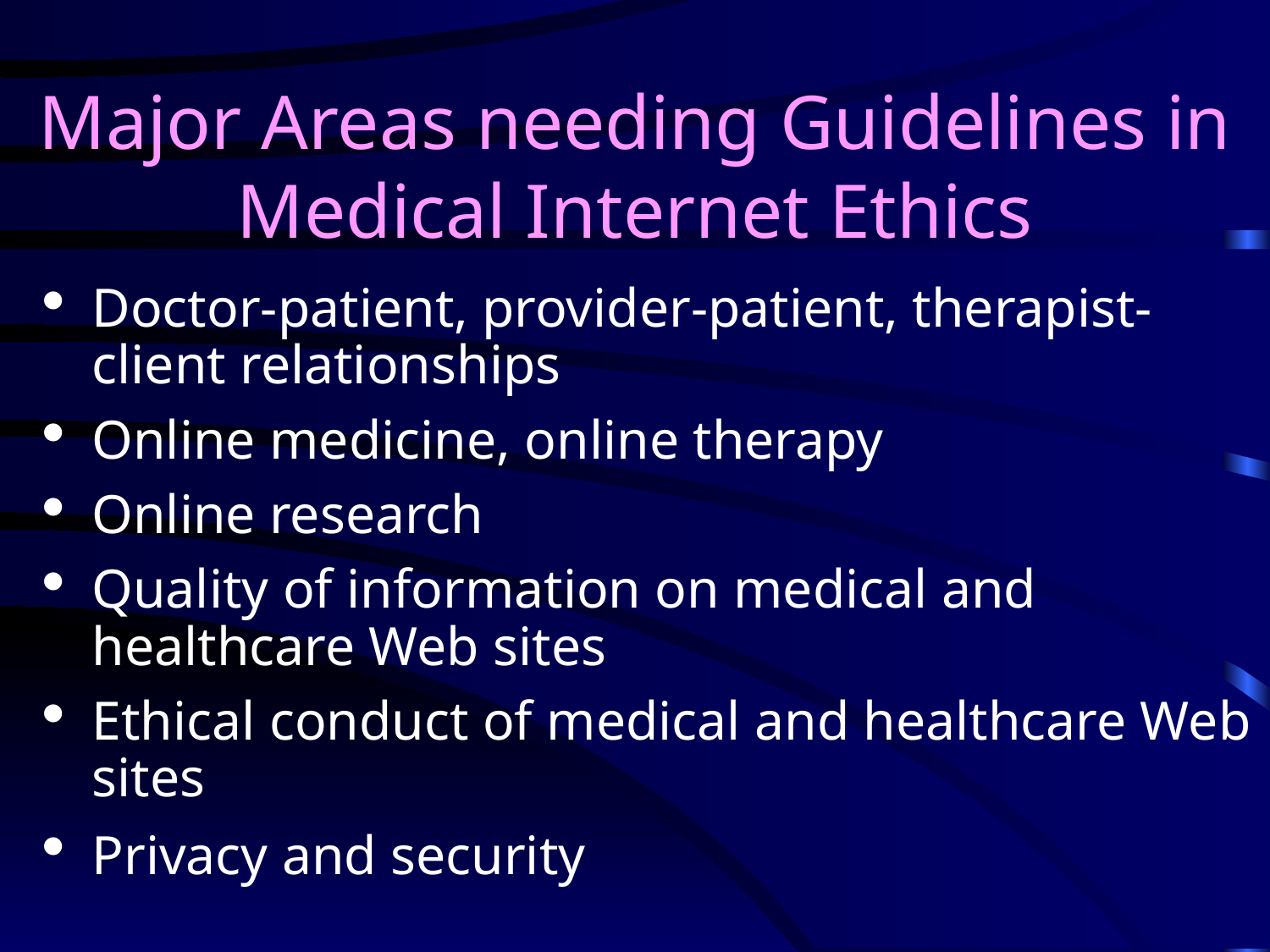

# Major Areas needing Guidelines in Medical Internet Ethics
Doctor-patient, provider-patient, therapist-client relationships
Online medicine, online therapy
Online research
Quality of information on medical and healthcare Web sites
Ethical conduct of medical and healthcare Web sites
Privacy and security

## Slide 12
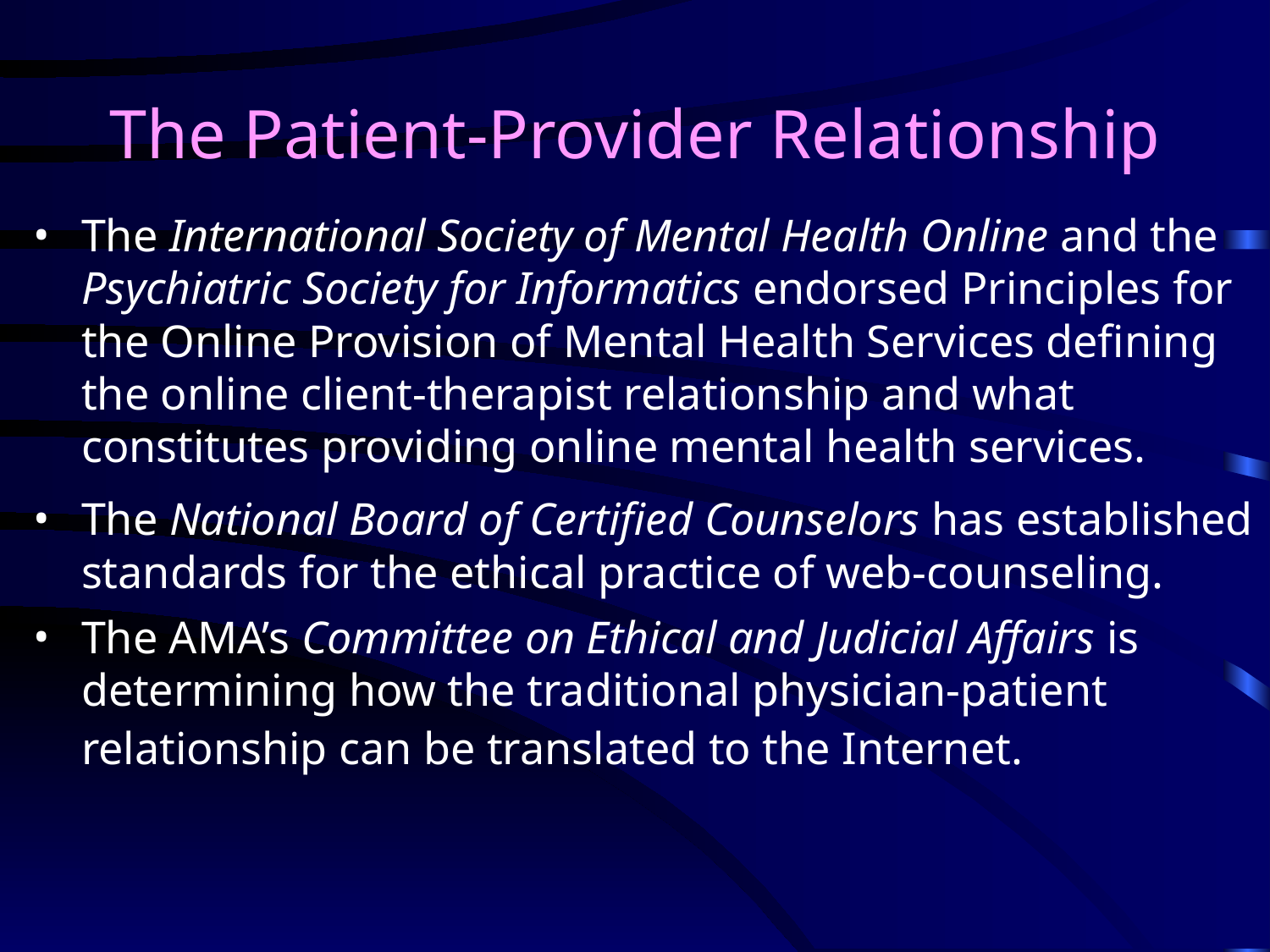

# The Patient-Provider Relationship
The International Society of Mental Health Online and the Psychiatric Society for Informatics endorsed Principles for the Online Provision of Mental Health Services defining the online client-therapist relationship and what constitutes providing online mental health services.
The National Board of Certified Counselors has established standards for the ethical practice of web-counseling.
The AMA’s Committee on Ethical and Judicial Affairs is determining how the traditional physician-patient relationship can be translated to the Internet.

## Slide 13
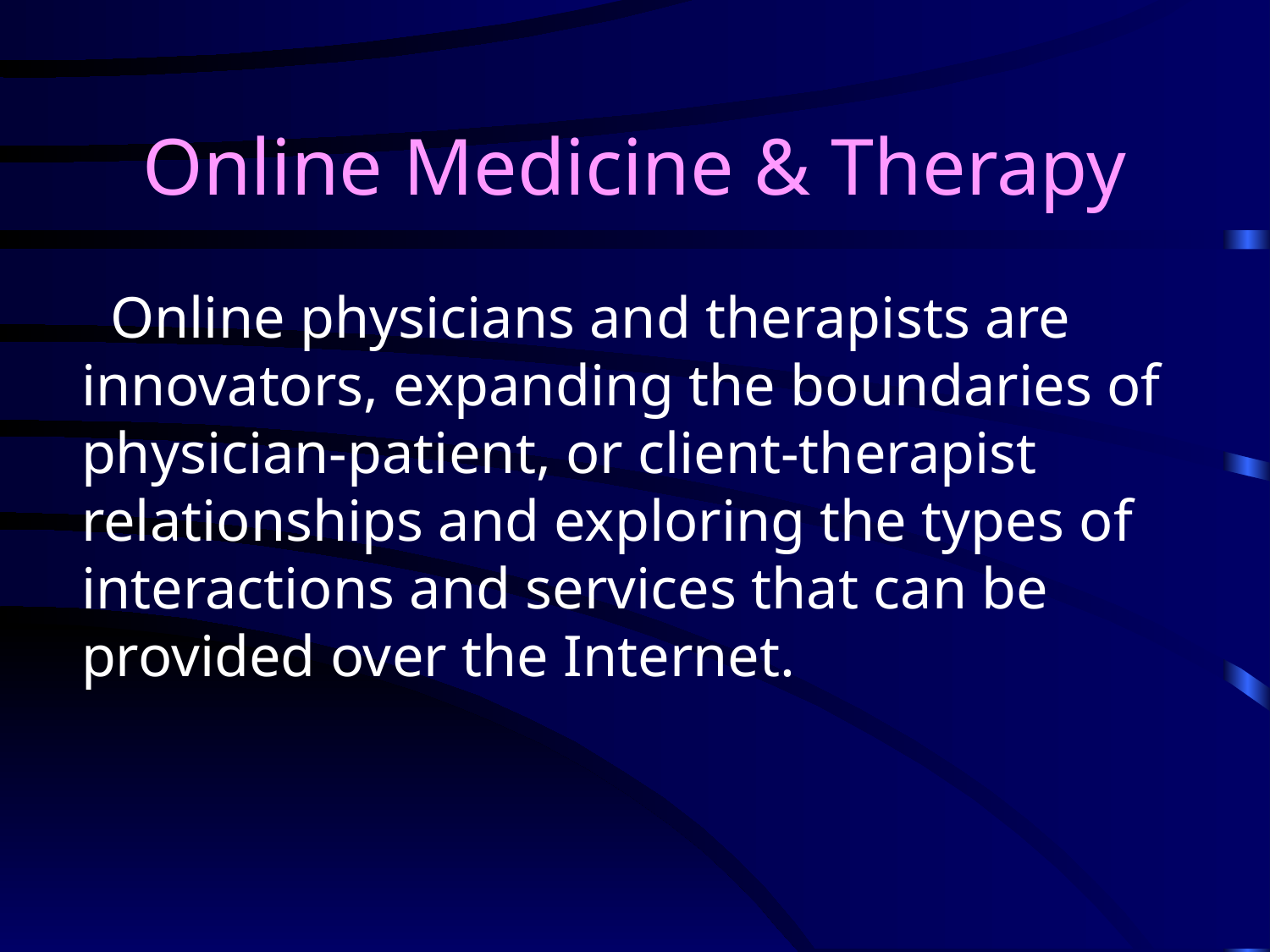

# Online Medicine & Therapy
 Online physicians and therapists are innovators, expanding the boundaries of physician-patient, or client-therapist relationships and exploring the types of interactions and services that can be provided over the Internet.

## Slide 14
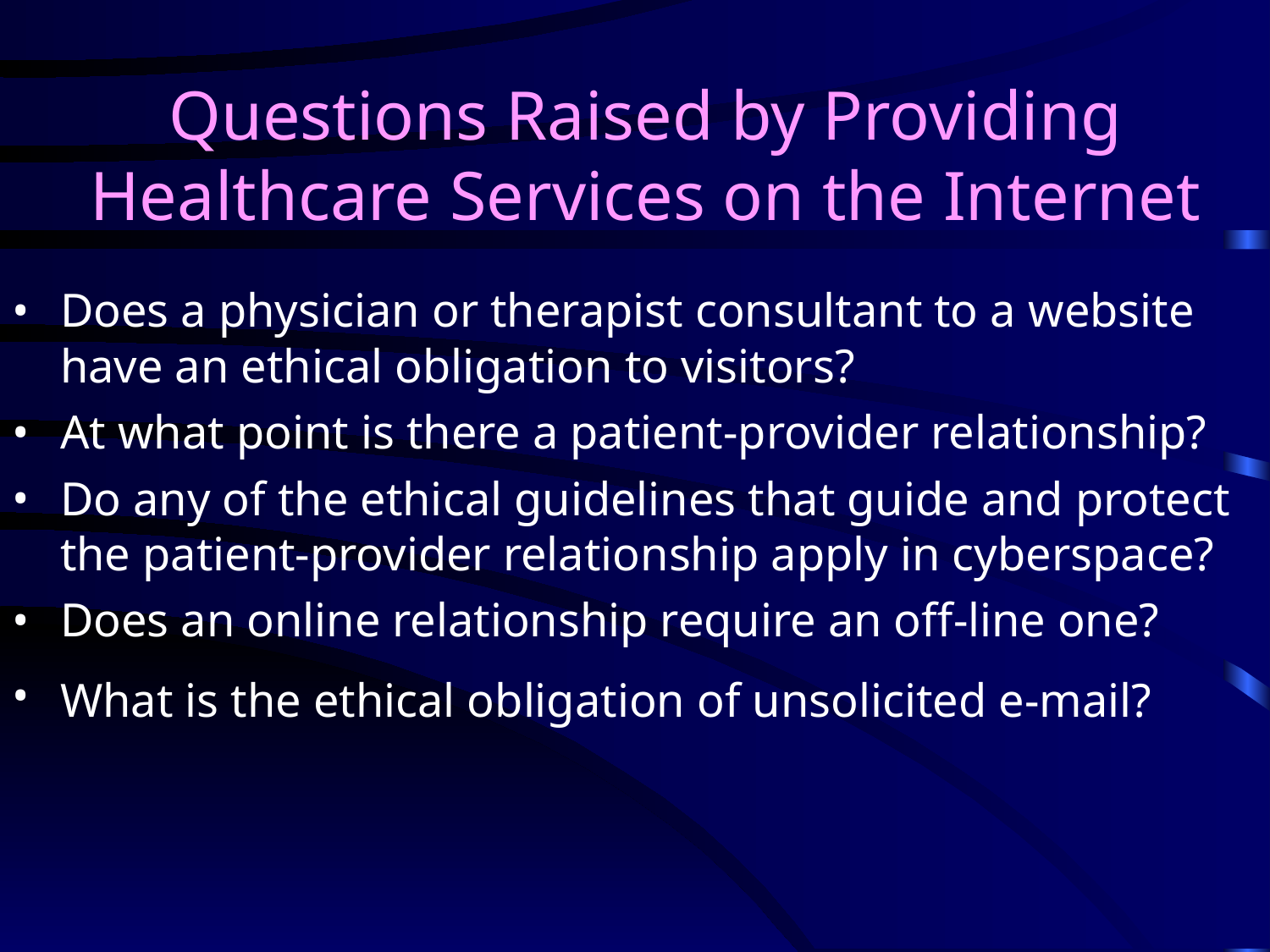

# Questions Raised by Providing Healthcare Services on the Internet
Does a physician or therapist consultant to a website have an ethical obligation to visitors?
At what point is there a patient-provider relationship?
Do any of the ethical guidelines that guide and protect the patient-provider relationship apply in cyberspace?
Does an online relationship require an off-line one?
What is the ethical obligation of unsolicited e-mail?

## Slide 15
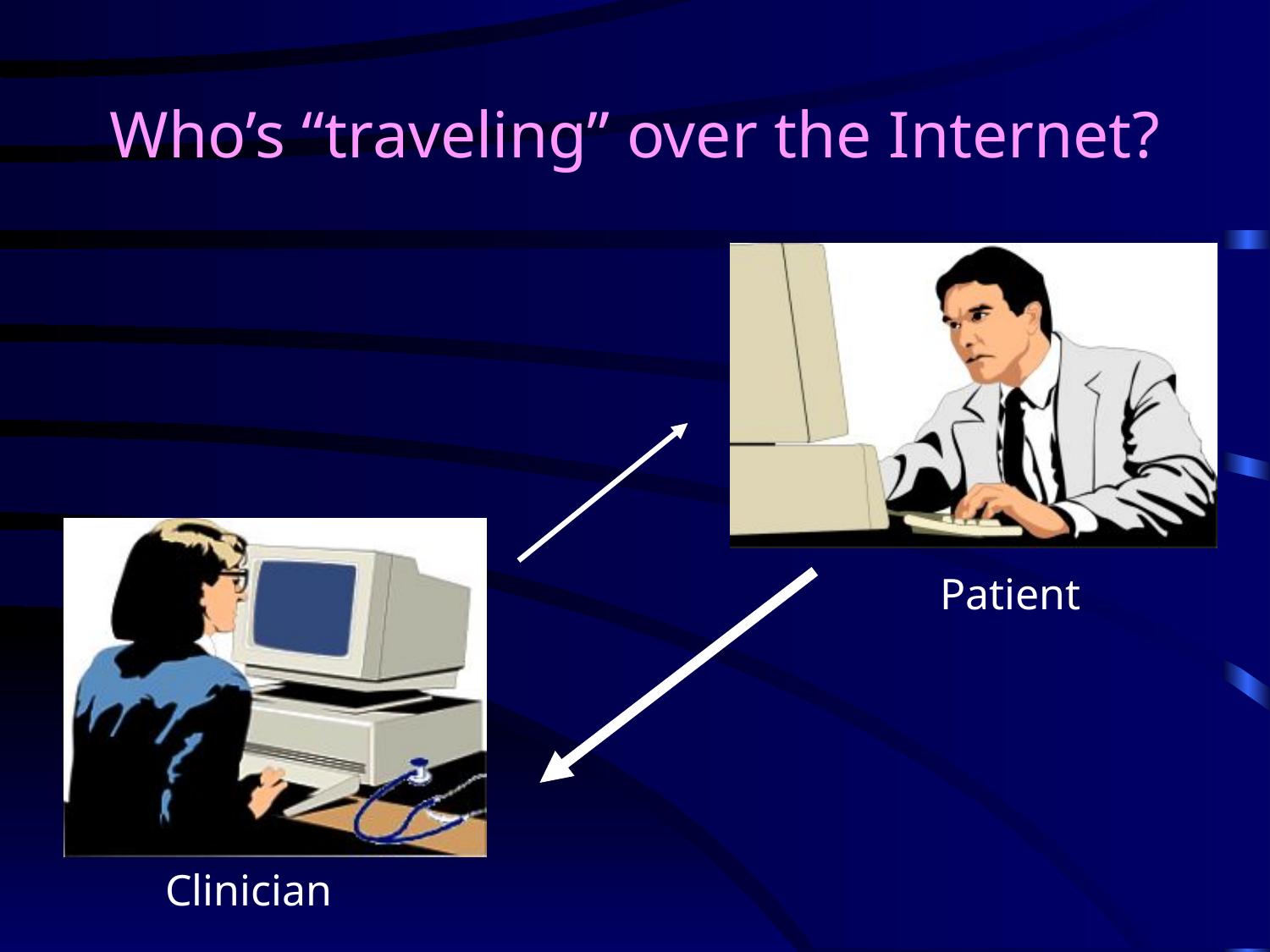

# Who’s “traveling” over the Internet?
Patient
Clinician

## Slide 16
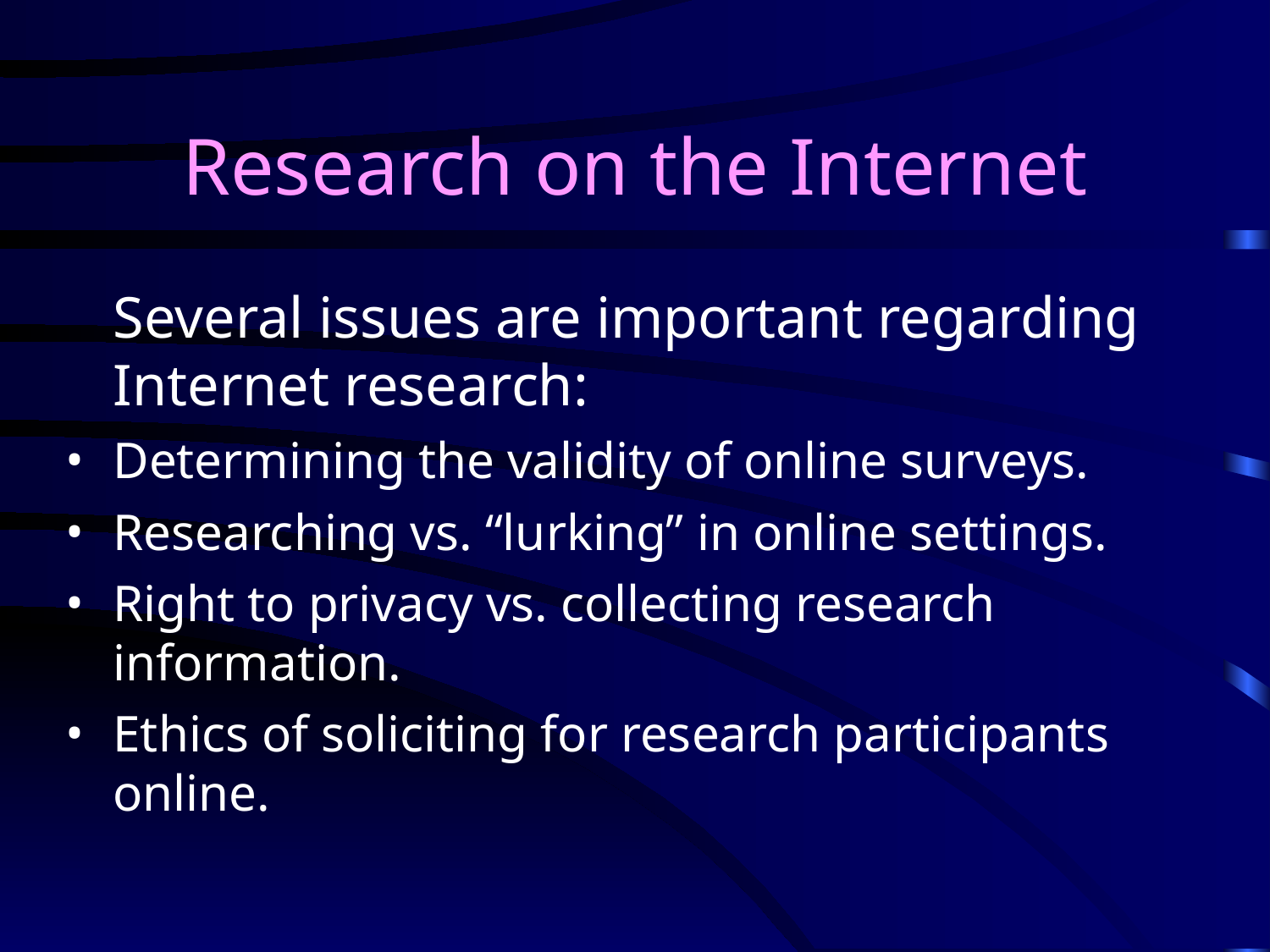

# Research on the Internet
Several issues are important regarding Internet research:
Determining the validity of online surveys.
Researching vs. “lurking” in online settings.
Right to privacy vs. collecting research information.
Ethics of soliciting for research participants online.

## Slide 17
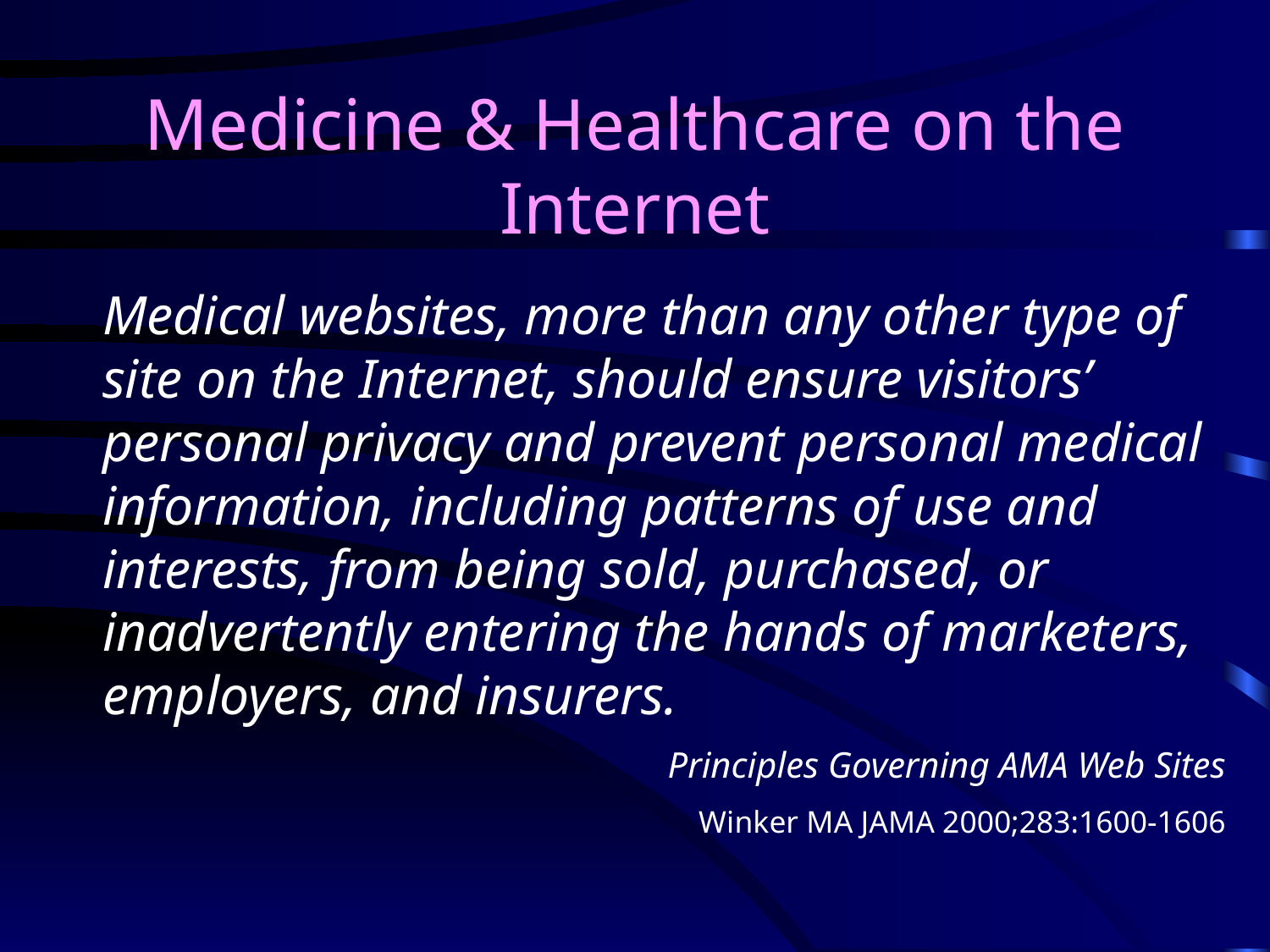

# Medicine & Healthcare on the Internet
Medical websites, more than any other type of site on the Internet, should ensure visitors’ personal privacy and prevent personal medical information, including patterns of use and interests, from being sold, purchased, or inadvertently entering the hands of marketers, employers, and insurers.
Principles Governing AMA Web Sites
Winker MA JAMA 2000;283:1600-1606

## Slide 18
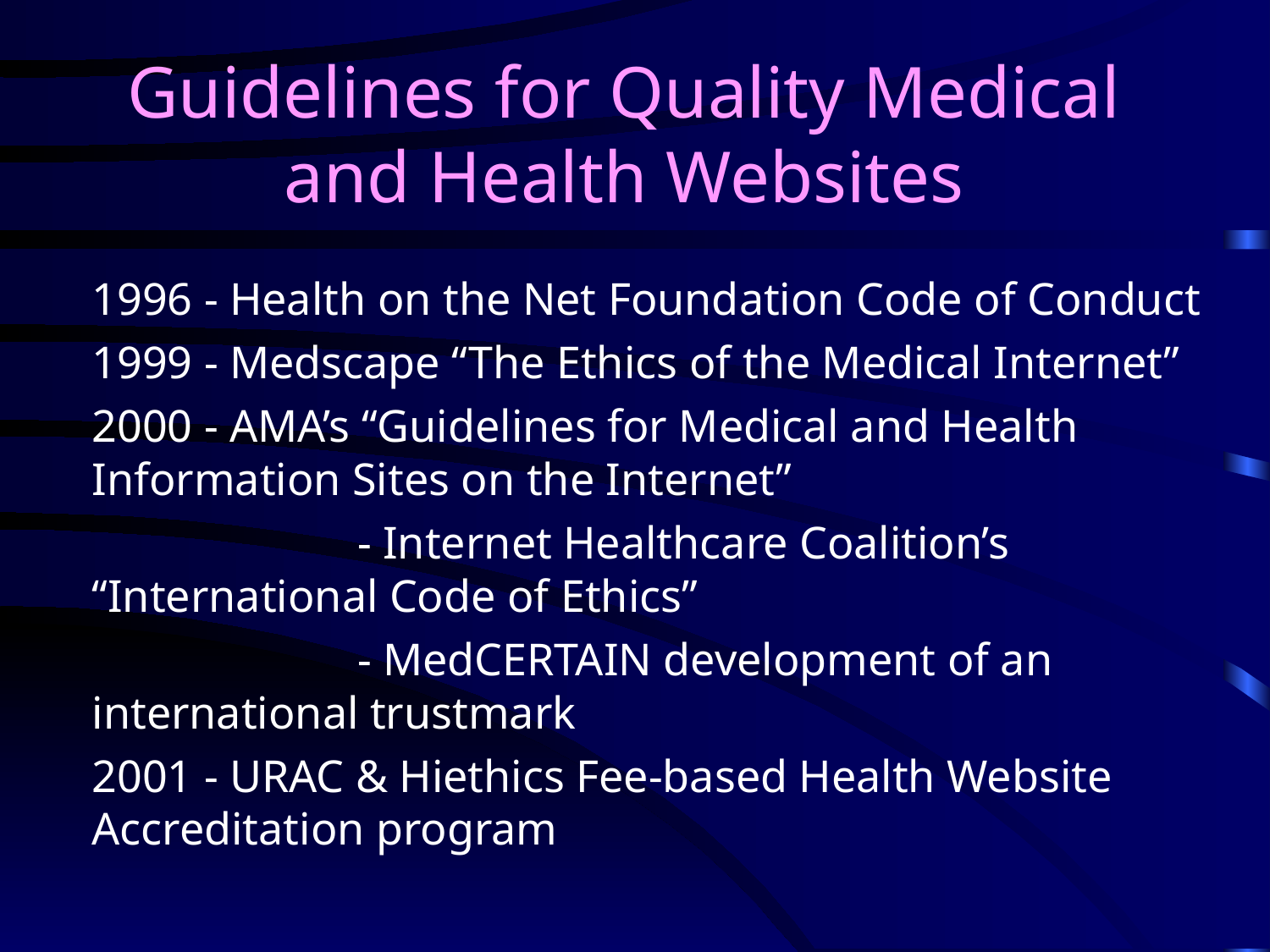

# Guidelines for Quality Medical and Health Websites
1996 - Health on the Net Foundation Code of Conduct
1999 - Medscape “The Ethics of the Medical Internet”
2000 - AMA’s “Guidelines for Medical and Health Information Sites on the Internet”
		 - Internet Healthcare Coalition’s “International Code of Ethics”
		 - MedCERTAIN development of an international trustmark
2001 - URAC & Hiethics Fee-based Health Website 	Accreditation program

## Slide 19
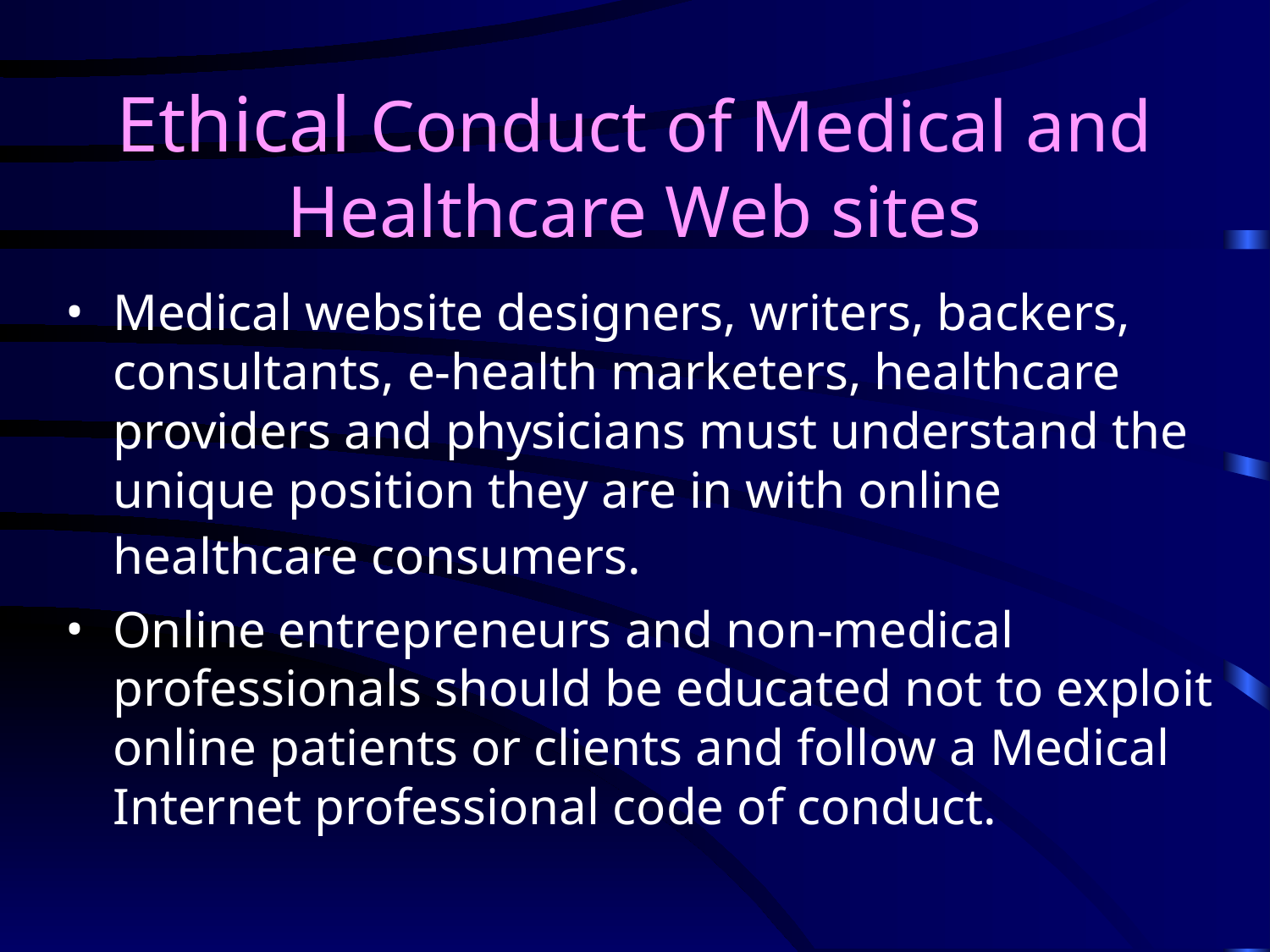

# Ethical Conduct of Medical and Healthcare Web sites
Medical website designers, writers, backers, consultants, e-health marketers, healthcare providers and physicians must understand the unique position they are in with online healthcare consumers.
Online entrepreneurs and non-medical professionals should be educated not to exploit online patients or clients and follow a Medical Internet professional code of conduct.

## Slide 20
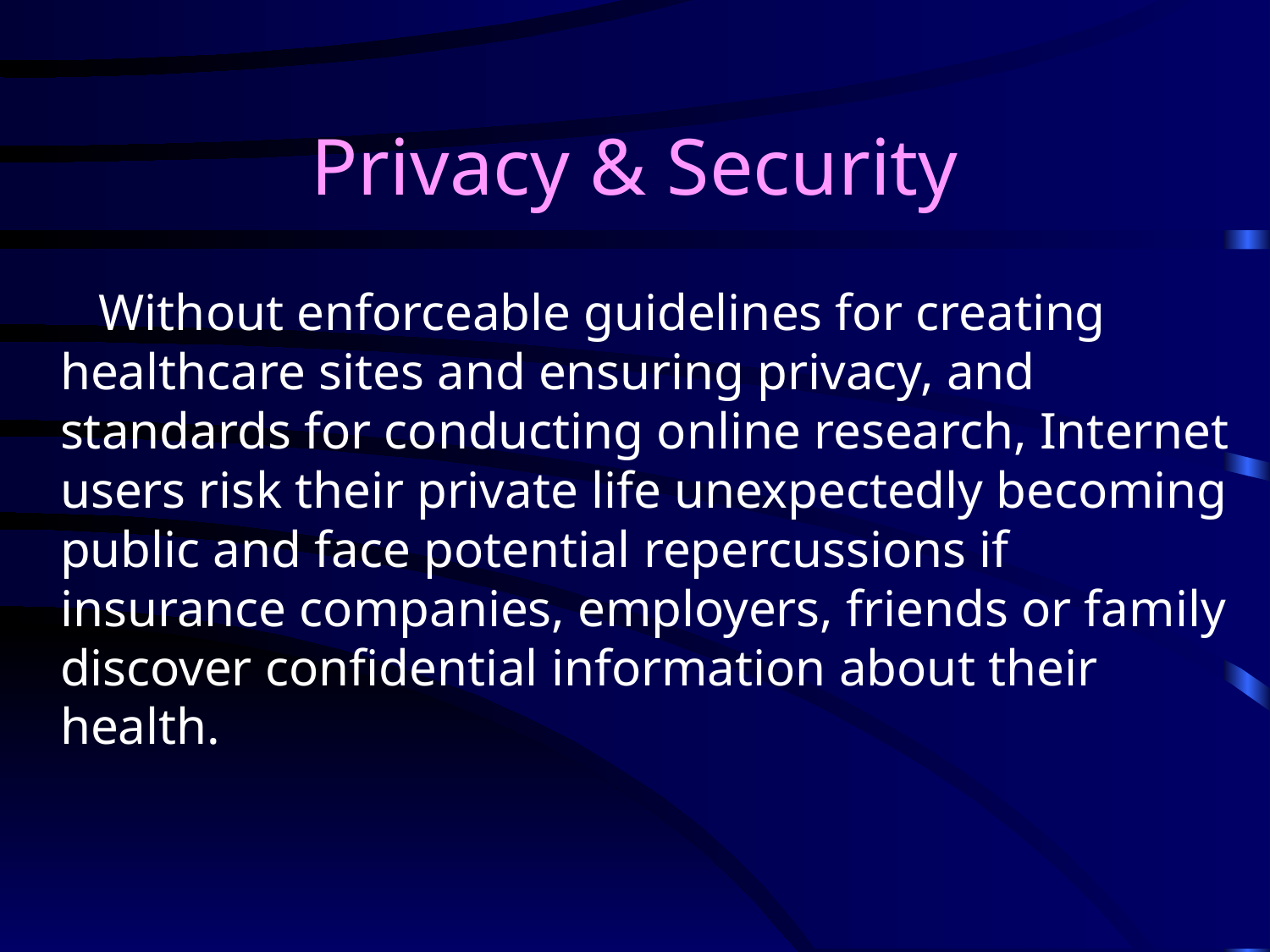

# Privacy & Security
 Without enforceable guidelines for creating healthcare sites and ensuring privacy, and standards for conducting online research, Internet users risk their private life unexpectedly becoming public and face potential repercussions if insurance companies, employers, friends or family discover confidential information about their health.

## Slide 21
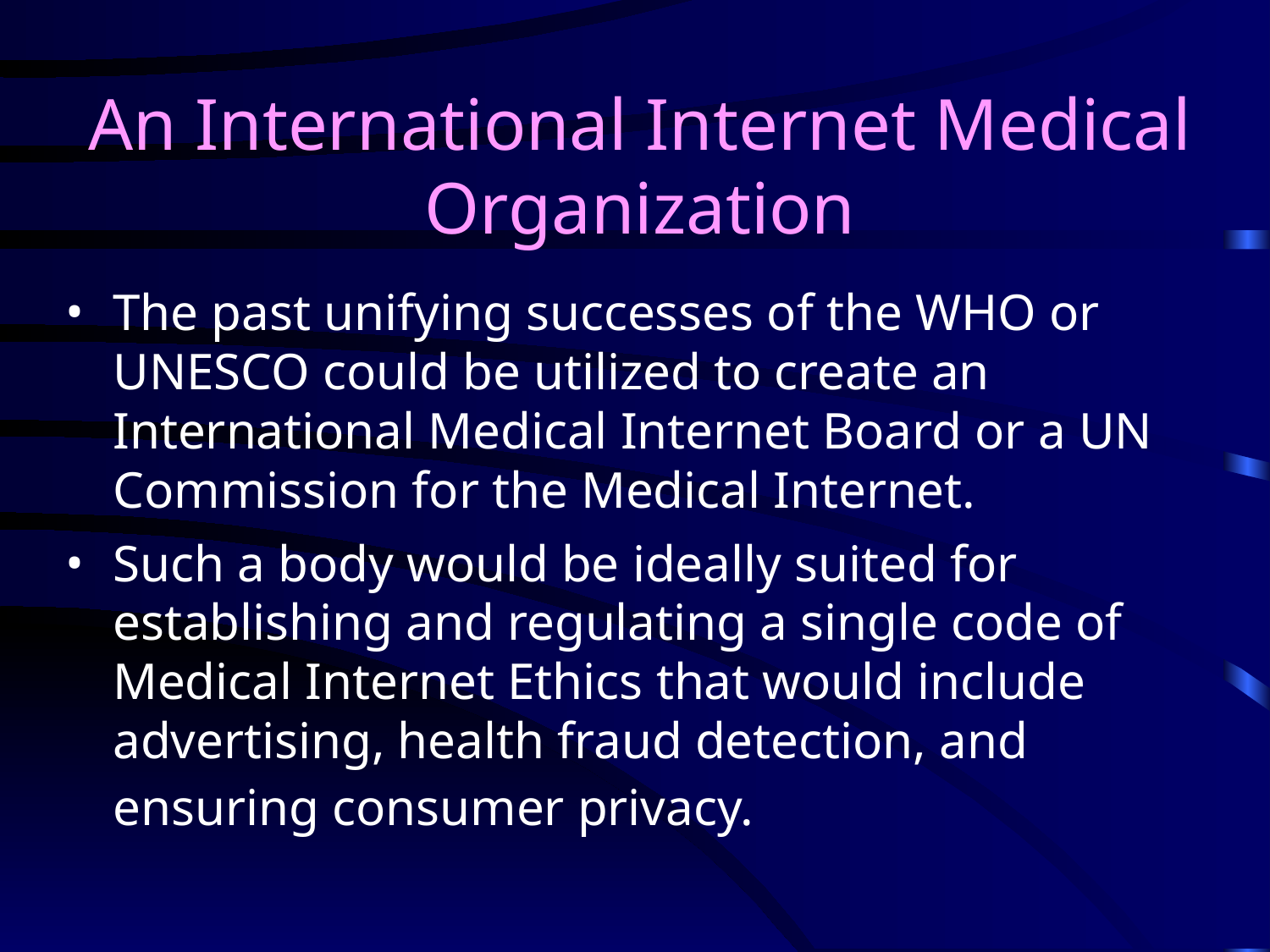

# An International Internet Medical Organization
The past unifying successes of the WHO or UNESCO could be utilized to create an International Medical Internet Board or a UN Commission for the Medical Internet.
Such a body would be ideally suited for establishing and regulating a single code of Medical Internet Ethics that would include advertising, health fraud detection, and ensuring consumer privacy.

## Slide 22
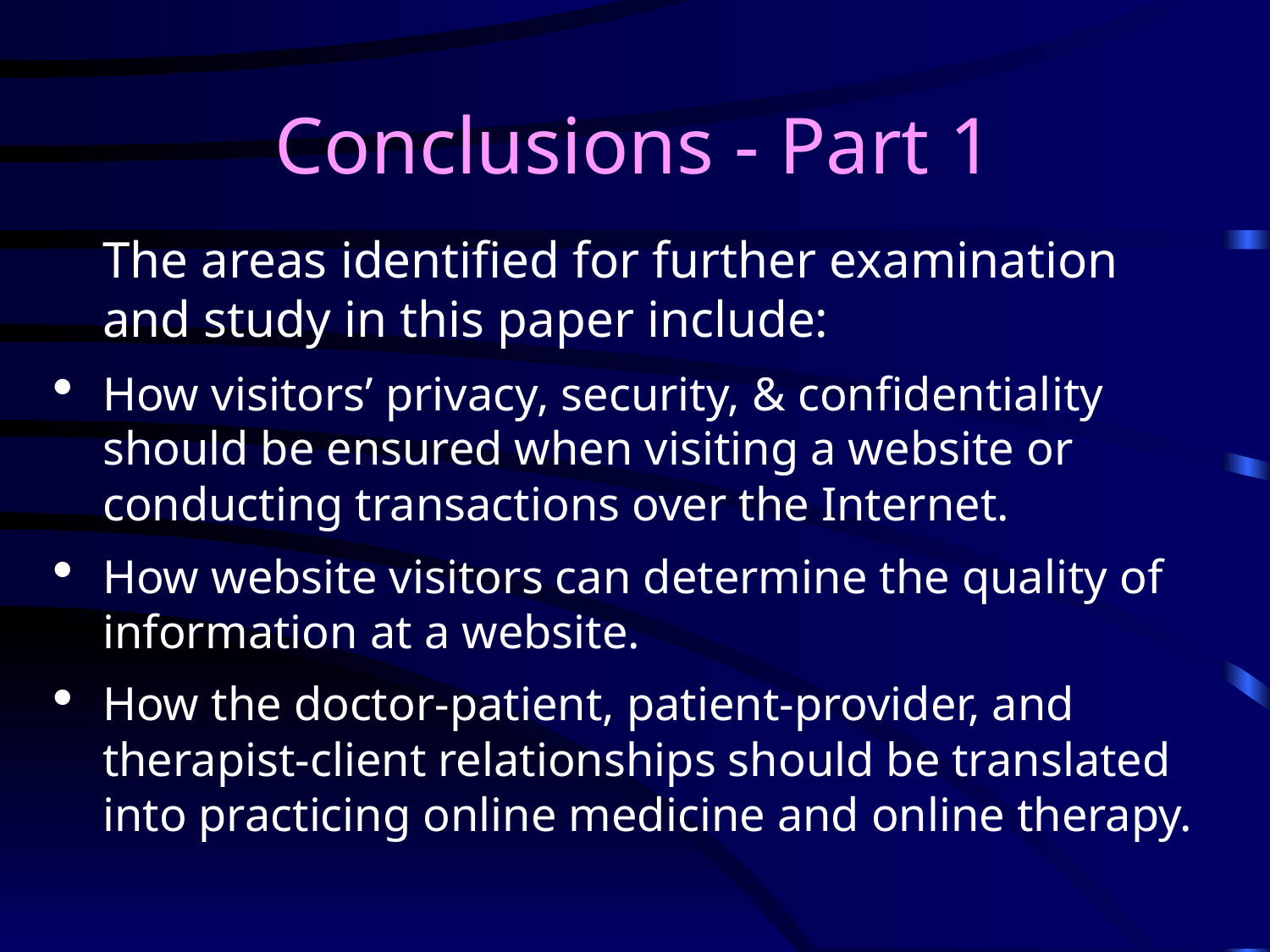

# Conclusions - Part 1
The areas identified for further examination and study in this paper include:
How visitors’ privacy, security, & confidentiality should be ensured when visiting a website or conducting transactions over the Internet.
How website visitors can determine the quality of information at a website.
How the doctor-patient, patient-provider, and therapist-client relationships should be translated into practicing online medicine and online therapy.

## Slide 23
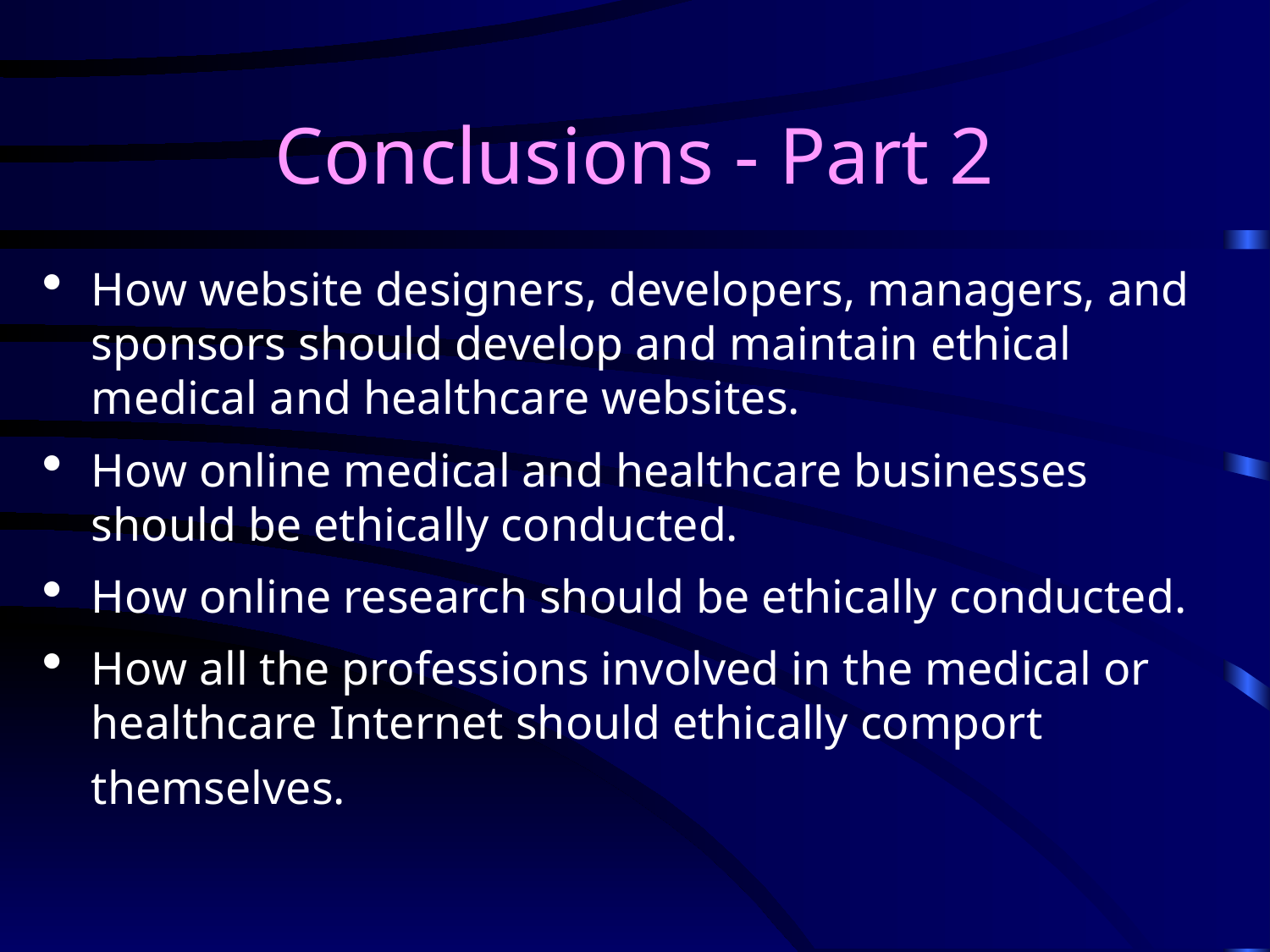

# Conclusions - Part 2
How website designers, developers, managers, and sponsors should develop and maintain ethical medical and healthcare websites.
How online medical and healthcare businesses should be ethically conducted.
How online research should be ethically conducted.
How all the professions involved in the medical or healthcare Internet should ethically comport themselves.

## Slide 24
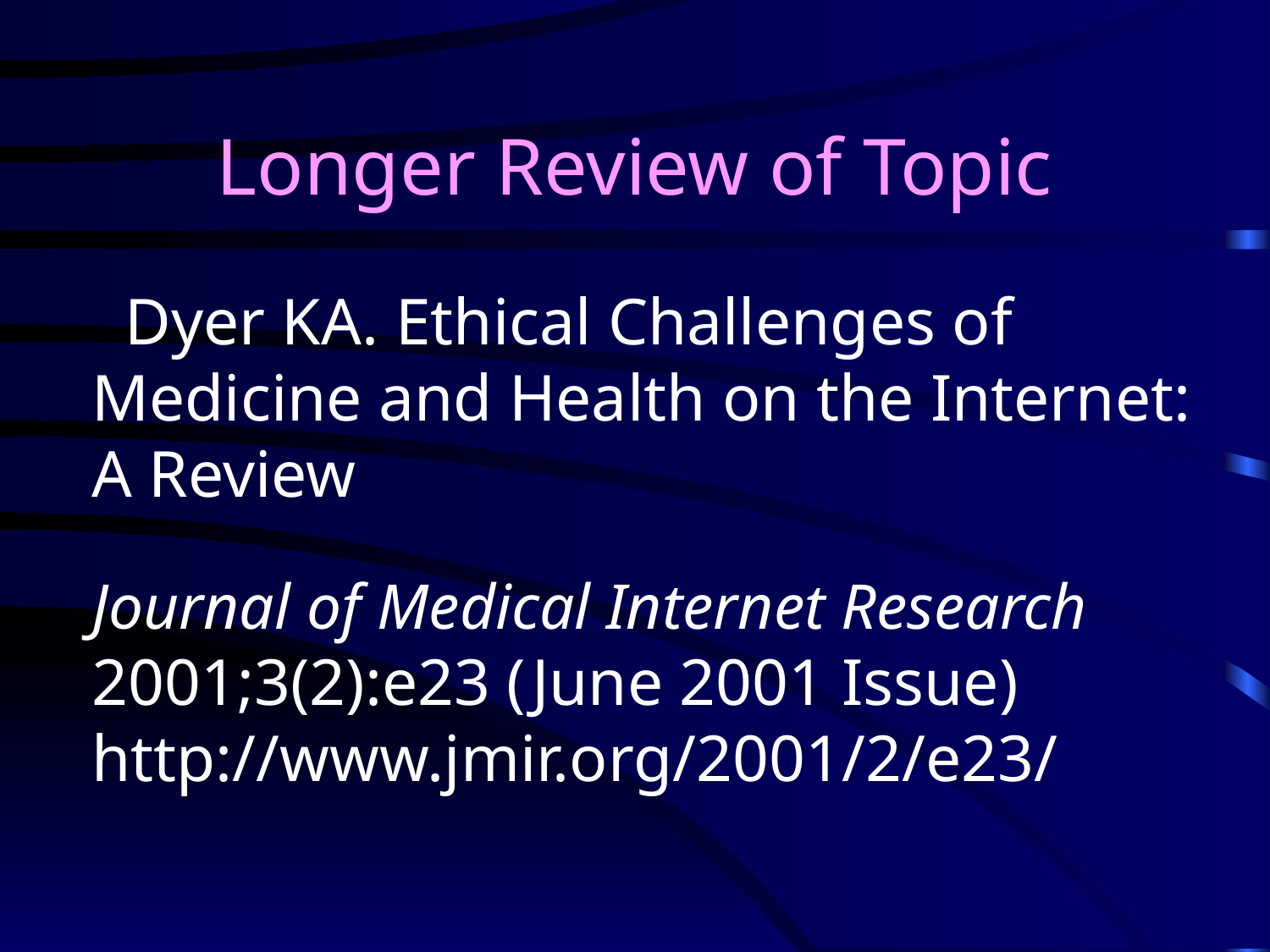

# Longer Review of Topic
 Dyer KA. Ethical Challenges of Medicine and Health on the Internet: A Review
Journal of Medical Internet Research 2001;3(2):e23 (June 2001 Issue)
http://www.jmir.org/2001/2/e23/
